# Deep learning for detection of age-related macular degeneration: A systematic review and meta-analysis of diagnostic test accuracy studies

**DOI:** 10.1371/journal.pone.0284060

**Published:** 2023-04-06

**Authors:** Xiangjie Leng, Ruijie Shi, Yanxia Wu, Shiyin Zhu, Xingcan Cai, Xuejing Lu, Ruobing Liu

**Affiliations:** 1 Eye College, Chengdu University of Traditional Chinese Medicine, Chengdu, Sichuan, China; 2 Department of Ophthalmology, Ineye Hospital of Chengdu University of Traditional Chinese Medicine, Chengdu, Sichuan, China; 3 Department of Ophthalmology, Key Laboratory of Sichuan Province Ophthalmopathy Prevention & Cure and Visual Function Protection with TCM Laboratory, Chengdu, Sichuan, China; 4 Department of Ophthalmology, Retinal Image Technology and Chronic Vascular Disease Prevention & Control and Collaborative Innovation Center, Chengdu, Sichuan, China; 5 Faculty of Technology, Policy and Management, Delft University of Technology, Delft, South Holland, Netherlands; Karunya Institute of Technology and Sciences, INDIA

## Abstract

**Objective:**

To evaluate the diagnostic accuracy of deep learning algorithms to identify age-related macular degeneration and to explore factors impacting the results for future model training.

**Methods:**

Diagnostic accuracy studies published in PubMed, EMBASE, the Cochrane Library, and ClinicalTrails.gov before 11 August 2022 which employed deep learning for age-related macular degeneration detection were identified and extracted by two independent researchers. Sensitivity analysis, subgroup, and meta-regression were performed by Review Manager 5.4.1, Meta-disc 1.4, and Stata 16.0. The risk of bias was assessed using QUADAS-2. The review was registered (PROSPERO CRD42022352753).

**Results:**

The pooled sensitivity and specificity in this meta-analysis were 94% (*P* = 0, 95% CI 0.94–0.94, *I*^*2*^ = 99.7%) and 97% (*P* = 0, 95% CI 0.97–0.97, *I*^*2*^ = 99.6%), respectively. The pooled positive likelihood ratio, negative likelihood ratio, diagnostic odds ratio, and the area under the curve value were 21.77(95% CI 15.49–30.59), 0.06 (95% CI 0.04–0.09), 342.41 (95% CI 210.31–557.49), and 0.9925, respectively. Meta-regression indicated that types of AMD (*P* = 0.1882, RDOR = 36.03) and layers of the network (P = 0.4878, RDOR = 0.74) contributed to the heterogeneity.

**Conclusions:**

Convolutional neural networks are mostly adopted deep learning algorithms in age-related macular degeneration detection. Convolutional neural networks, especially ResNets, are effective in detecting age-related macular degeneration with high diagnostic accuracy. Types of age-related macular degeneration and layers of the network are the two essential factors that impact the model training process. Proper layers of the network will make the model more reliable. More datasets established by new diagnostic methods will be used to train deep learning models in the future, which will benefit for fundus application screening, long-range medical treatment, and reducing the workload of physicians.

## Introduction

Age-related macular degeneration (AMD) is one of the leading causes of severe irreversible vision impairment in developed countries [1, 2]. With the accelerated aging process of the global population, the number of AMD patients is expected to increase to 288 million by 2040 [3], and it has become one of the key topics in the research of ophthalmic blindness prevention.

Clinically, it is classified as dry AMD (dAMD) characterized by medium-sized drusen and retinal pigmentary changes, and wet AMD (wAMD) characterized by neovascular and atrophic [4]. Fundus photography (FP) and optical coherence tomography (OCT) are the most widely used auxiliary examinations in ophthalmology. FP is the cheapest and the most necessary fundus test in AMD, which can intuitively identify lesions and diagnose AMD. OCT uses low coherence light to scan biological tissues in cross-section and converts the acquired information into numbers. After computer processing, it displays the pathological changes of each layer of the retina clearly and provides quantitative diagnostic indicators. In addition, OPTOS ultra-widefield retinal images can clearly visualize peripheral retinal lesions, and when combined with angiography, it can clearly show peripheral choroidal neovascularization (CNV) [5], and produces better pseudocolor images than conventional 45° FP in diagnosis [6]. AMD first affects the retinal pigment epithelium, Bruch’s membrane, and choroidal capillaries in the macular area. AMD can be manifested as drusen, atrophy of the outer retinal structure, CNV, polypoid lesions, and pigment epithelial detachment in OCT images.

The rapid increase in the demand for screening and follow-up of AMD means that a large number of human and financial resources need to be provided by the healthcare systems of various countries. The use of deep learning (DL) model technology may be a long-term solution for screening and monitoring patients in primary eye care settings.

The DL model is a branch of machine learning, composed of neural networks that are good at computer vision, perception, and image recognition. DL model uses multilayer nonlinear information processing modules to extract supervised or unsupervised features from a set of training data and make the correct prediction. In recent years, DL models have been widely used in ophthalmology [7–9], dermatology [10], radiology [11, 12], pathology [13, 14], and many other image-centric specialties. In ophthalmology-related research, DL models are beginning to be widely used in the diagnosis and recognition of diseases including diabetic retinopathy [15–17], AMD [15, 18–20], glaucoma [21], refractive error [22], and prematurity retinopathy of prematurity [23–25].

To establish a DL system, technical network and the datasets are the most essential components. Although not all CNN (Convolutional Neural Network) belongs to deep learning, CNN is the most widely used technical network in AMD diagnostic research which can operate on the whole images without requiring radiologists or ophthalmologists to manually contour on images [26]. A CNN can be divided into input, hidden, and output layers. The hidden layers are usually composed of convolutional, pooling, full connection, and normalization layers. The core of the CNN is the convolutional layer, which transforms the input data by applying a set of filters (also known as kernels) that act as feature detectors. A CNN learns the values of these filters’ weights on its own during the training process [27]. Activations are used after convolution. The pooling layers can reduce the dimensionality and keep the most important information. The output of the convolutional and pooling layers represents high-level features of the input image. The purpose of the fully connected layer is to use these high-level features to classify the input image categories based on the training dataset. Afterwards, backpropagation is performed to calculate the network weights, and gradient descent is used to update all filters and parameter values to minimize the output error [27]. This process will be repeated many times.

The datasets for AMD detection are various. Most public databases were established using FP and OCT images. Peking University collected a structured FP database of 5,000 patients including normal, diabetes, glaucoma, cataract, AMD, hypertension (H), myopia, and other diseases/abnormalities in 2019. The database is named as Ocular Disease Intelligent Recognition (ODIR) [28]. iChallenge-AMD is composed of AMD and non-AMD (myopia, normal control, etc.) FPs [29]. Srinivasan et al [30] conducted an OCT database (Duke dataset) that was acquired from 45 patients: 15 normal patients, 15 patients with dry AMD, and 15 patients with DME in 2014. Established by Rasti et al [31] in 2017, the Noor dataset was acquired at Noor Eye Hospital in Tehran and is consisting of 50 normal, 48 dAMD, and 50 DME OCTs. Regarding the Kaggle dataset [32], OCT images were selected from retrospective cohorts of adult patients from the Shiley Eye Institute of the University of California San Diego, the California Retinal Research Foundation, Medical Center Ophthalmology Associates, the Shanghai First People’s Hospital, and Beijing Tongren Eye Center between July 1, 2013 and March 1, 2017. Kermany et al [33] established an OCT database (Mendeley dataset) that contains CNV, DME, Drusen and normal people in 2018. Gholami et al [34] established an AMD retinal OCT images database including 55 AMD images called OCTID (Optical Coherence Tomography Image Database) in 2019. Besides public datasets, plenty of studies choose self-built databases which obtained data from hospitals directly.

DL models, especially CNN, have flourished rapidly in AMD detection in recent years. Although most of the DL models show effective diagnostic accuracy, DL specialists are still trying to explore the best networks, diameters, and layers of the network for higher accuracy. This meta-analysis summarized the DL models for AMD diagnosis and aimed to evaluate the diagnostic accuracy of DL models and to explore the best settings for future AMD model training, which will benefit researchers interested in DL for the diagnosis of fundus disorder.

## Methods

This systematic review and meta-analysis was conducted according to the Preferred Reporting Items for Systematic Reviews and Meta-Analysis of Diagnostic Test Accuracy Studies (PRISMA-DTA) [35], and the Cochrane handbook [36]. The PRISMA-DTA checklists are available in **S1 and S2 Tables**. This meta-analysis was registered on PROSPERO (ID: CRD42022352753).

### Eligibility criteria

All peer-reviewed and preprint original articles that reported the sensitivity and specificity of DL models in detecting AMD were considered. The detailed inclusion criteria were as follows: (1) diagnosing AMD by DL model via various images; (2) true positive (TP), false positive (FP), true negative (TN), and false negative (FN) could be obtained or transferred from the study. Records without available data such as reviews, conference abstracts, letters, and replies were excluded. There was no restriction on the year of publication, language, country, or datasets.

### Information sources, search strategy and study selection

The search engines used included PubMed, EMBASE, the Cochrane Library, Web of Science, Scopus, ScienceDirect ClinicalTrails.gov, and World Health Organization International Clinical Trial Registration Platform (WHO ICTRP), and Chinese Clinical Trail Registry (ChiCTR) by 11 August 11, 2022.

The search strategy using medical subject headings (MeSH and Emtree) combined with entry words for all search engines. Detailed search strategies in different search engines are detailed in **S1 File**.

Endnote 20 was adopted for the study selection process. Duplicate studies were excluded by automation tools. The titles and abstracts were independently identified for possible inclusion by two authors (Leng X. and Shi R.). Disagreements were resolved by a third researcher (Wu Y.). After full text selection, the reports assessed eligibility were included in this meta-analysis.

### Data collection process and definitions for data extraction

The data from the included studies were extracted by an individual researcher (Cai X.) and were rechecked by another (Zhu S.). The data we extracted included the first author and published year, country, number of images, network layers, device, hardware, type of AMD datasets, total dataset size, type of images, TP, FP, FN, TN, AUC, sensitivity, and specificity.

AMD, including dAMD and wAMD, was considered as the target condition. The reference standard was clinically proven AMD, while the DL-based diagnosis was considered the index test.

### Risk of bias and applicability

The study risk of bias assessment was conducted by two individual researchers using the QUADAS-2 (Quality Assessment of Diagnostic Accuracy Studies-2) tool (Leng X., Shi R.). Parameters included patient selection, index test, reference standard, flow and timing, and applicability concerns in terms of patient selection, index test, and reference standard. Disagreements were solved with consensus by the third researcher (Lu X.). Deeks’ funnel plot mapped by Stata 16.0 was applied to assess the potential publication bias. An asymmetrical funnel shape or a *P* < 0.05 means the presentation of publication bias [37].

### Diagnostic accuracy measures and synthesis of results

To evaluate the diagnostic accuracy of deep learning in detecting AMD, the sensitivity, specificity, positive likelihood ratio (PLR), negative likelihood ratio (NLR), and diagnostic odds ratio (DOR) along with a 95% Confidence Interval (CI) were calculated separately for each study. Random effects models were applied in the calculation of the pooled results.

### Meta-analysis and additional analysis

Separate and summary results of sensitivity and specificity would be presented in a form of a forest plot. The heterogeneity of the meta-analysis was evaluated by the Cochran Q-test and *I*^*2*^ [38]. *I*^*2*^ exceeding 25%, 50% and 75% indicate the meta-analysis with low, medium, and high heterogeneity respectively [39]. Sensitivity analyses, subgroup analyses, and meta-regression were conducted to explore the sources of heterogeneity. All meta-analyses and additional analyses were performed using Metadisc 1.4 and Review manager 5.4.1.

## Results

### Study selection

**Fig 1:**
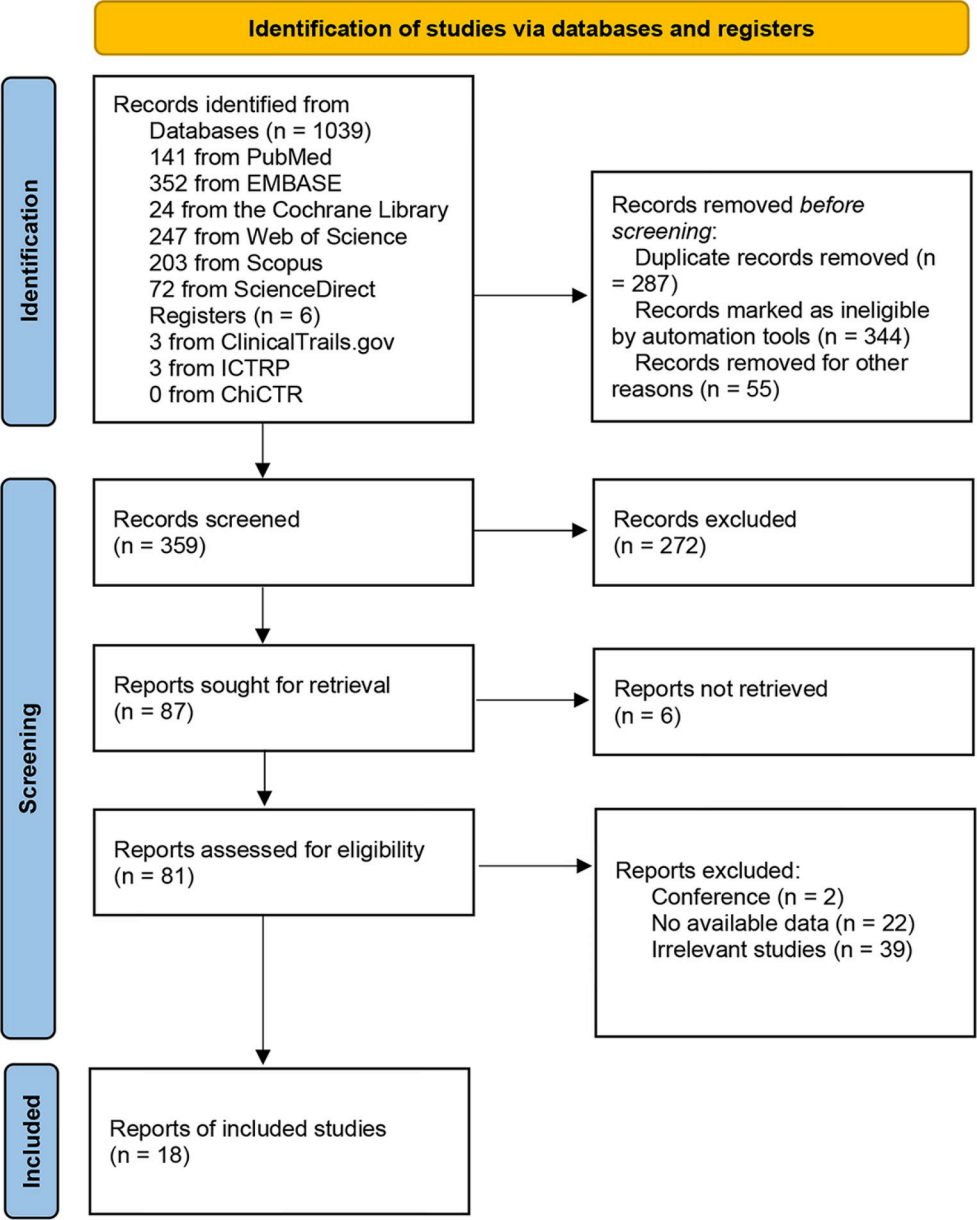
Study selection flow diagram.

The detailed study selection process is described in **Fig 1**. 1045 records were searched using the present search strategy. 359 records remained after eliminating duplicate records and the ineligible records marked by automation tools. 272 records were excluded by screening titles and abstracts. 87 reports were sought for retrieval, of which 6 reports were not retrieved. 81 reports were assessed for eligibility through full text reading, and 2 conference abstracts, 39 irrelevant studies, and 22 no-available-data studies were excluded. Finally, 18 eligible studies were extracted from the remaining articles by full text review.

### Study characteristics

The detailed studies characteristics are summarized in **Table 1**. The 18 studies included were reported as full-text articles which consist of 56 models and summarized data from 778052 various images. OCT images, FPs, OPTOS ultra-widefield retinal images, and OCT images combined with FP images were included in 10, 5, 1, and 3 studies, respectively. All studies were published in 2017–2022, which were conducted in China, the USA, Japan, India, Jordan, the United Kingdom, Turkey, Russia, South Korea, Singapore, Norway and Spain. As for the variants, 5 studies used VGG, and 5 studies used ResNet. CapsNet, Darknet and other networks such as AlexNet, DenseNet, and self-created networks were adopted in one study, respectively. The layers of the network were divided into five classes including ≤10, 10–20, 20–50, 50–100, and >100, which were adopted in 2, 10, 6, 2 and 1 studies, respectively.

**Table 1:** Characteristics of included studies.

First author & published year	Country	Variant of CNN	Layers	GPU	Datasets	Number of Images	Images	Other images in datasets	AMD
Alqudah 2019 [40]	Jordan	Self-created	19	Nvidia Tesla K40 (12GB)	Duke, Mendeley, Duke, Self-built	135596	OCT	CNV, DME, Normal	All
Bhatia 2019 [41]	UK	VGG	16	NA	Mendeley, Noor, Self-built	5588	OCT	CNV, DME, Normal	All
Celebi 2022 [42]	Turkey	CapsNet	7	Nvidia Tesla K40 (12GB)	Kaggle dataset, Self-built	726	OCT	Normal	All
Dong 2022 [43]	China	Darknet	53	NA	Multicenter Self-built	208758	FP	Normal	All
Gour 2020 [44]	India	VGG	16	NA	ODIR	331	FP	Cataract, Diabetes, Glaucoma, Hyperattention, Myopia, other abnormalities, Normal	All
He 2022 [45]	China	ResNet	50	NA	Duke, Mendeley	795	OCT	DME, Normal	All
Kadry 2021 [46]	Norway	VGG	16	NA	iChallenge-AMD database, OCTID	6400	OCT, FP	Non-AMD	All
			19						
		AlexNet	11						
		ResNet	50						
Lee 2017 [47]	USA	VGG	16	NVIDIA Pascal Titan X (12GB)	Self-built	101002	OCT	Normal	All
Ma 2022 [48]	USA	ResNet	34	Nvidia V100 (32 GB)	Self-built	73	OCT	PCV	Wet
Mathews 2022 [49]	India	Self-created	11	NA	Duke, Mendeley	75	OCT	DME, Normal	Dry
Matsuba 2019 [50]	Japan	DCNN	7	NA	Self-built	364	OPTOS^2^	Normal	Wet
Motozawa 2019 [51]	Japan	Unclear	18	GTX 1080 TI (11GB)	Self-built	169	OCT	Normal	All
Takhchidi 2021 [52]	Russia	ResNet	50	Nvidia RTX 2070 Max-Q (8GB)	Self-built	1200	FP	Normal	All
Tan 2018 [53]	Singapore	Unclear	14	NA	Self-built	1110	FP	Normal	All
Thomas 2021 [54]	India	Unclear	19	Nvidia RTX2080 (8GB)	Duke, Mendeley, Noor, OCTID	1139	OCT	Normal	All
Wang 2019 [55]	China	DenseNet	121	NVIDIA RTX 2080 TI (11G)	Duke, Noor	8315	OCT	DME, Normal	All
		ResNet	50						
		ResNext	101						
		DPN	92						
		CliqueNet	10						
Yoo 2018 [56]	Korea	VGG	19	NVIDIA GTX1060 (3GB); GTX980 (6GB)	Project Macula	83	OCT, FP	Normal	All
Zapata 2020 [57]	Spain	Self-created	24	NA	Optretina’s tagged dataset	306302	OCT, FP	GON^3^	All

NA, not applicable; OPTOS, OPTOS ultra-widefield retinal images; GON, glaucomatous optic neuropathy

### Risk of bias and bias of publication

**Fig 2:**
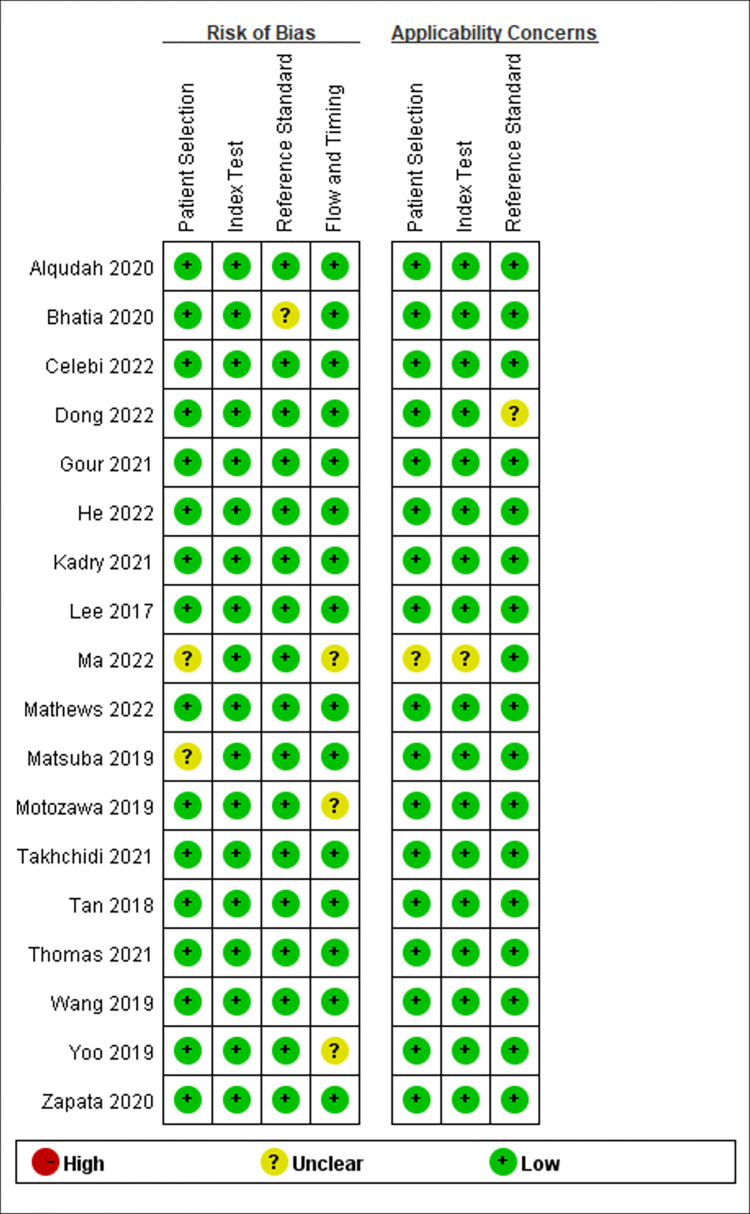
QUADAS-2 results in each study.

The results of the QUADAS-2 analysis are summarized in **Fig 2**. Generally, the risk of bias is low for this meta-analysis. The risk of patient selection was considered “low risk” in 16 studies and “unclear risk” in 2 studies. The risk of bias for the index test and reference standard was “low risk” in all studies. The risk of bias for reference standard was rated “low” in 17 studies and 1 were rated “unclear risk”. The risk of bias for flow and timing was rated “low” in 15 studies and 3 were rated “unclear risk”. Applicability concerns including patient selection, index test, and the reference standard only existed in one “unclear risk” study and the other 17 studies were rated “low risk”.

**Fig 3:**
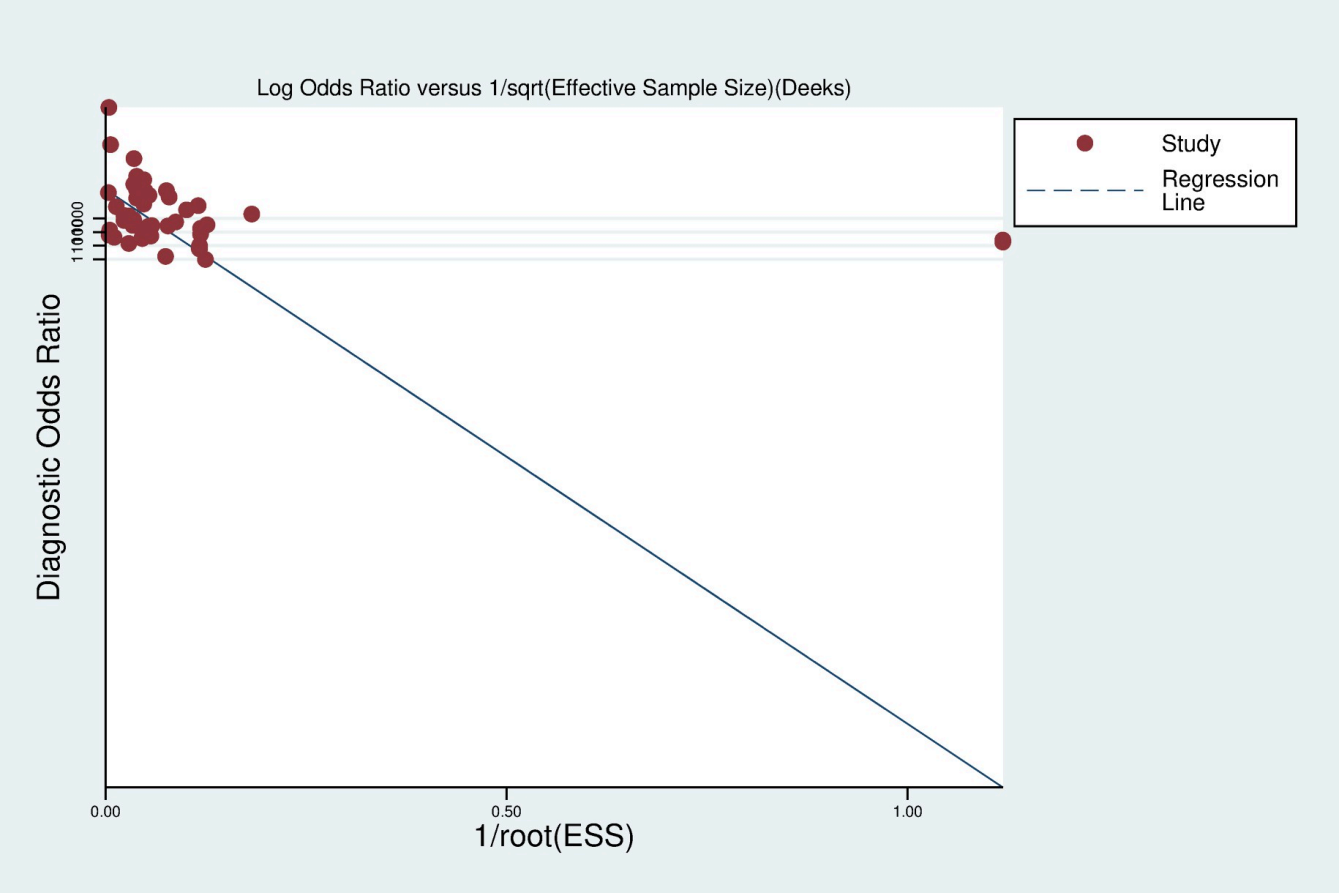
Deek’s funnel plot.

Deeks’ funnel plot (**Fig 3**) was adopted to investigate the potential bias of publication by Stata 16.0 (*P* = 0.375, 95%CI -292.9264 to 112.091), which indicated no obvious publication bias existed in this meta-analysis.

### Results of individual studies

In this research, various DL models were mentioned in the included studies for AMD identification, including VGG, CapsNet, ResNet, AlexNet, DenseNet, ResNext, DPN, CliqueNet etc. The results show that these models have high sensitivity and specificity in AMD identification, which can meet the needs of practical clinical applications. The detailed results of individual studies are summarized in **Table 2**. Alqudah et al [40] used a 15-layer CNN to classify 136,187 OCT images from Mendeley, Duke, and Self-built datasets (4 classes including AMD, CNV, DME, and normal) for AMD identifying with a sensitivity of 100% and a specificity of 100%. Bhatia et al [41] used VGG-16 to classify 5588 OCT images from Mendeley, Duke, Noor, and Self-built datasets (4 classes including AMD, CNV, DME, normal) for AMD identifying with a sensitivity of 94% and a specificity of 90%. Celebi et al [42] used CapsNet with 7 layers to classify 726 OCT images form Kaggle and self-built datasets (2 classes including AMD and normal) for AMD identifying with a sensitivity of 100% and a specificity of 99%. Dong et al [43] used a joint CNN detector using Yolov3 to classify 208758 FP images from self-built multicenter real-world data (11 classes including AMD, DR, glaucoma, pathological myopia, retinal vein occlusion, macula hole, epiretinal macular membrane, hypertensive retinopathy, myelinated fibers, retinitis pigmentosa and normal) for AMD identifying with a sensitivity of 88% and a specificity of 98%. Gour et al [44] used VGG-16 to classify 331 FP images from ODIR dataset (8 classes including AMD, cataract, diabetes, glaucoma, hyperattention, myopia, and other abnormalities) for AMD identifying with a sensitivity of 6% and a specificity of 94%. He et al [45] used ResNet-50 to classify 795 OCT images from Mendeley and Duke datasets (3 classes including AMD, DME, and normal) for AMD identifying with a sensitivity of 96% and a specificity of 99%. Kadry et al [46] used VGG-16, VGG-19, AlexNet, and ResNet-50 to classify 3200 FP images and 3200 OCT images from iChallenge AMD database, OCTID (2 classes including AMD and Non-AMD) resulting in sensitivity of 88%, 84%, 88%, 88% and specificity of 85%, 87%, 85%, 84%, respectively. Lee et al [47] used VGG-16 to classify 101002 OCT images from self-built dataset (2 classes including AMD and normal) for AMD identifying with a sensitivity of 90% and a specificity of 91%. Ma et al [48] used ResNet-34 to classify 73 OCT images from self-built dataset (2 classes including AMD and polypoidal choroidal vasculopathy) for AMD identifying with a sensitivity of 92% and a specificity of 90%. Mathews et al [49] used a 11-layer lightweight CNN to classify 75 OCT images from Duke and Mendeley datasets (3 classes including AMD, DME, and normal) for AMD identifying with a sensitivity of 100% and a specificity of 100%. Matsuba et al [50] used a 7-layer CNN to classify 364 OPTOS ultra-widefield retinal images from self-built dataset (2 classes including AMD and normal) for AMD identifying with a sensitivity of 100% and a specificity of 97%. Motozawa et al [51] used an 18-layer CNN to classify 169 OCT images from self-built database (2 classes including AMD and normal) for AMD identifying with a sensitivity of 99% and a specificity of 100%. Takhchidi et al [52] used ResNet-50 to classify 1200 FP images from self-built dataset (2 classes including AMD and normal) for AMD identifying with a sensitivity of 90% and a specificity of 86%. Tan et al [53] used a 14-layer CNN to classify 1110 FP images from self-built dataset (2 classes including AMD and normal) for AMD identifying with a sensitivity of 96% and a specificity of 94%. Thomas et al [54] used a 14-layer CNN to classify 1139 OCT images from Mendeley, Duke, Noor, and OCTID datasets (2 classes including AMD and normal) for AMD identifying with a sensitivity of 99% and a specificity of 100%. Wang et al [55] used DenseNet, ResNet, ResNext, DPN, and CliqueNet to classify 8315 OCT images from Duke and Noor datasets (3 classes including AMD, DME and normal) resulting in sensitivity of 96%, 97%, 100%, 97%, 99% and specificity of 95%, 100%, 100%, 97%, 99% in dataset 1, and sensitivity of 95%, 100%, 99%, 100%, 93% and specificity of 95%, 99%, 95%, 99%, 98% in dataset 2. Yoo et al [56] used VGG-19 to classify three types of images (OCT, FP, and OCT combined with FP) from Project Macula (2 classes including AMD and normal) for AMD identifying with a pooled sensitivity of 84% and a pooled sensitivity of 59%. Zapata et al [57] used a 24-layer CNN to classify 306302 FP images and OCT images from Optretina’s tagged dataset (2 classes including AMD and glaucomatous optic neuropathy) for AMD identifying with a sensitivity of 83% and a specificity of 89%.

**Table 2:** Summary of each included studies.

Study	Methodology	Datasets	Number of images	Classes	Other diseases	Sensitivity	Specificity	Limitations
Alqudah 2019 [40]	15-layer CNN	Duke, Mendeley, Self-built	136187 OCT images	4	CNV, DME, Normal	100%	100%	
Bhatia 2019 [41]	VGG-16	Duke, Mendeley, Noor, Self-built	5588 OCT images	4	CNV, DME, Normal	94%	90%	1) Ignored bad quality pictures.
Celebi 2022 [42]	CapsNet	Kaggle dataset,	726 OCT images	2	Normal	100%	99%	1)Did not study other retinal diseases; 2)Ignored bad quality pictures and patients who had other retinal diseases.
Dong 2022 [43]	A joint CNN detector using Yolov3	Multicenter Self-built	208758 FP images	11	DR, Glaucoma, Pathological myopia, Retinal vein occlusion, Macula hole, Epiretinal macular membrane, Hypertensive retinopathy, Myelinated fibers, Retinitis pigmentosa, Normal	88%	98%	1)Only small number of retinitis pigmentosa.
Gour 2020 [44]	VGG-16	ODIR	331 FP images	8	Cataract, Diabetes, Glaucoma, Hyperattention, Myopia, other abnormalities, Normal	6%	94%	1)The dataset contained 8 types of diseases, but with a small dataset.
He 2022 [45]	ResNet-50	Duke, Mendeley	795 OCT images	3	DME, Normal	96%	99%	1)Only contained one other diseases.
Kadry 2021 [46]	VGG-16	iChallenge-AMD database, OCTID	3200 FP and 3200 OCT images	2	Non-AMD	88%	85%	1)The definition of non-AMD is not clear.
	VGG-19					84%	87%	
	AlexNet,					88%	85%	
	ResNet-50					88%	84%	
Lee 2017 [47]	VGG-16	Self-built	101002 OCT images	2	Normal	90%	91%	1)Included only images from patients who met the study criteria, and the neural network was only trained on these images; 2) This model was trained using images from a single academic center, and the external generalizability is unknown
Ma 2022 [48]	ResNet-34	Self-built	73 OCT images	2	Polypoidal choroidal vasculopathy	92%	90%	1) Small dataset
Mathews 2022 [49]	A 11-layer lightweight CNN	Duke, Mendeley	10907 OCT images	3	DME, Normal	100%	100%	1) This study used drusen macular degeneration for AMD diagnosis; 2)Only contain one other diseases.
Matsuba 2019 [50]	A 7-layer CNN	Self-built	364 OPTOS images	2	Normal	100%	97%	1) It is difficult to acquire precise images using OPTOS when the transmission of light into the eye is impaired by an intermediate translucent zone; 2) Most AMD patients accept treatment which may cause diagnostic error 3) Did not study other retinal diseases.
Motozawa 2019 [51]	An 18-layer CNN	Self-built	169 OCT images	2	Normal	99%	100%	1) Excluded low quality images and patients who had other concomitant diseases; 2) Did not study other retinal diseases.
Takhchidi 2021 [52]	ResNet-50	Self-built	1200 FP images	2	Normal	90%	86%	1) Did not study other retinal diseases.
Tan 2018 [53]	A 14-layer CNN	Self-built	1110 FP images	2	Normal	96%	94%	1) Did not study other retinal diseases.
Thomas 2021 [54]	A 19-layer CNN	Mendeley, Duke, Noor, OCTID	1139 OCT images	2	Normal	99%	100%	1) Did not study other retinal diseases.
Wang 2019 [55]	DenseNet-121	Duke, Noor	8315 OCT images	3	DME, Normal	96% in Duke, 95% in Noor	95% in Duke, 95% in Noor	1) Only contained one other diseases.
	ResNet-50					97% in Duke, 100% in Noor	100% in Duke, 99% in Noor	
	ResNext-101					100% in Duke, 99% in Noor	100% in Duke, 95% in Noor	
	DPN-92					97% in Duke, 100% in Noor	97% in Duke, 99% in Noor	
	CliqueNet-10					99% in Duke, 93% in Noor	99% in Duke, 98% in Noor	
Yoo 2018 [56]	VGG-19	Project Macula	83 FP and 83 OCT images	2	Normal	84%	59%	1) Did not study other retinal diseases; 2) Small datasets;
Zapata 2020 [57]	A 24-layer CNN	Optretina’s tagged dataset	306302 FP images and OCT images	2	Glaucomatous optic neuropathy	83%	89%	1.No clear number of OCT or FP images.

### Results of synthesis

**Fig 4:**
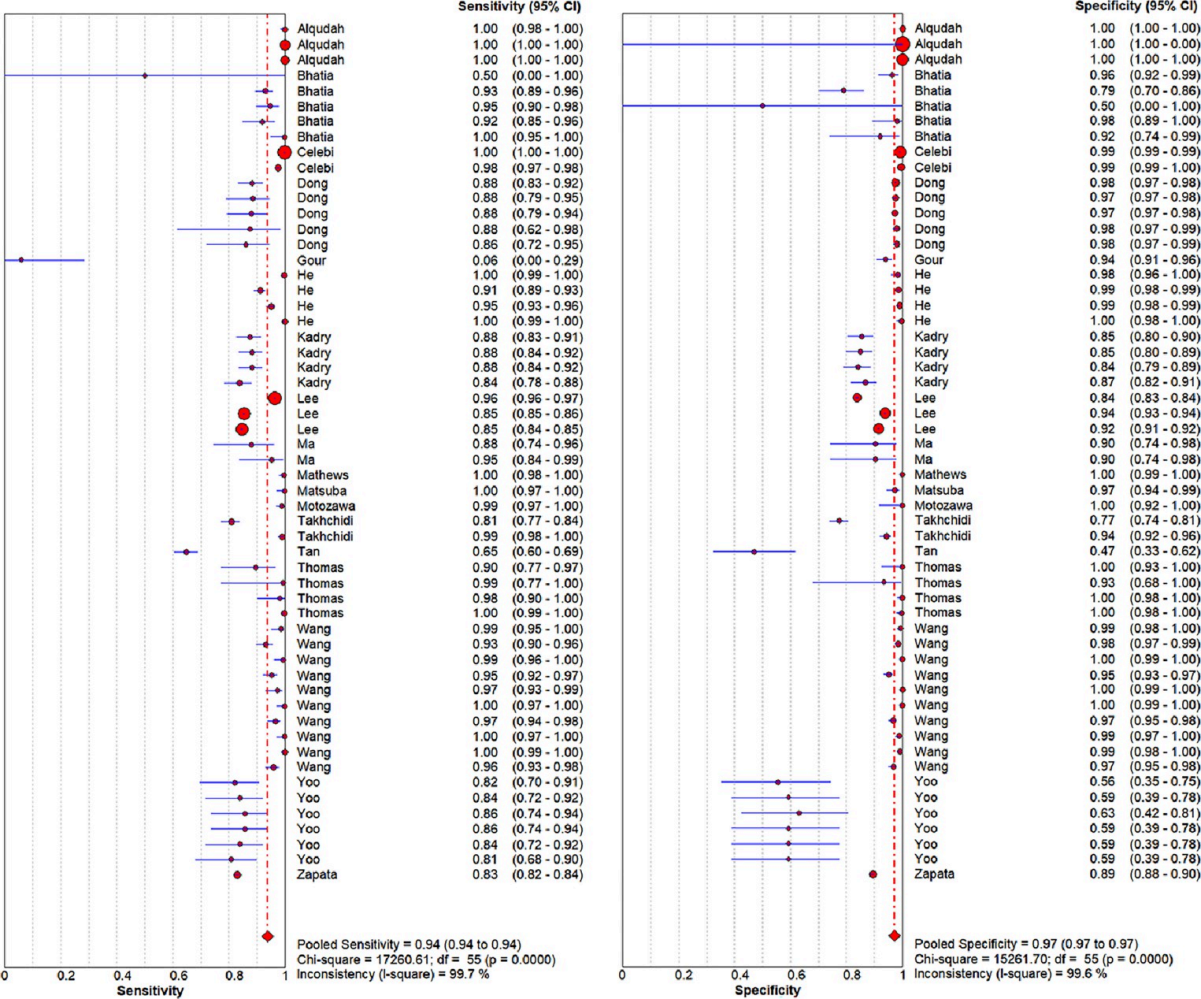
The forest plot of the pooled sensitivity and specificity.

**Fig 5:**
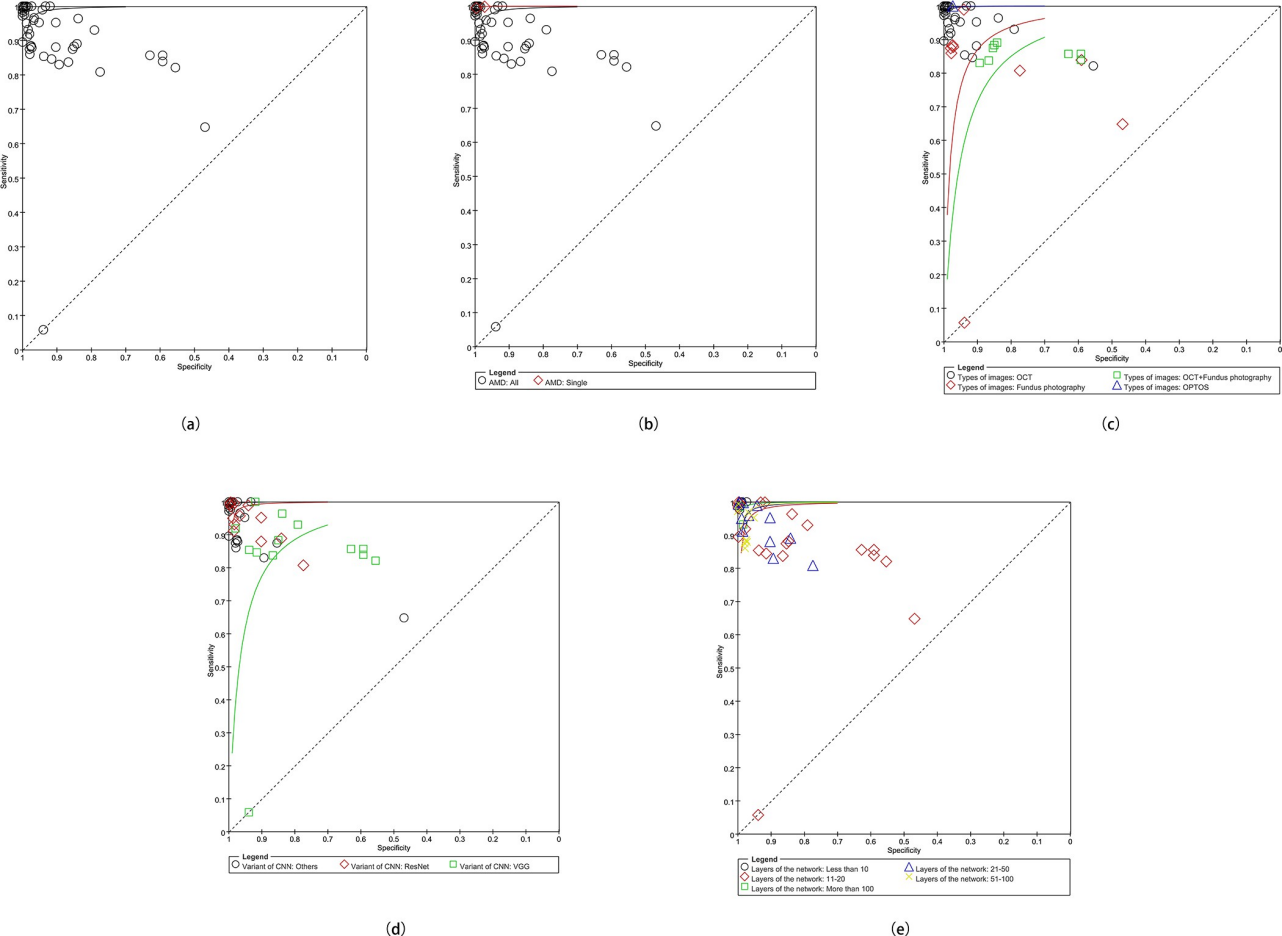
The SROC (a) the pooled SROC; (b) the SROC of types of AMD; (c) the SROC of types of images; (d) the SROC of variants of CNNs; (e) the SROC of Networks.

The pooled sensitivity and specificity in this meta-analysis were 94% (*P* = 0, 95% CI 0.94–0.94, *I*^*2*^ = 99.7%) and 97% (*P* = 0, 95% CI 0.97–0.97, *I*^*2*^ = 99.6%) (**Fig 4, S1 Fig),** respectively. The PLR, NLR, DOR, and AUC values were 21.77(95% CI 15.49–30.59), 0.06 (95% CI 0.04–0.09), 342.41 (95% CI 210.31–557.49) and 0.9925. The SROC (Summary Receiver Operating Characteristic) curves are showed in **Fig 5(A)**.

### Additional analysis

For the high heterogeneity, the additional analyses were conducted based on the results of sensitivity and specificity. Sensitivity analyses were conducted to investigate the sources of heterogeneity, however, neither the *I*^*2*^ of sensitivity nor specificity significantly decreased after excluding studies one by one. Therefore, subgroup analyses which included the type of AMD, type of images, variant of CNN, and variants were conducted (**Fig 5B–5E**). Meta regression indicated that the sources of heterogeneity were types of AMD (*P* = 0.1882, RDOR = 36.03) and layers of the network (*P* = 0.4878, RDOR = 0.74). All additional analyses results were summarized in **Table 3**. Original forest plots were available in **S1 Fig**.

**Table 3:** Subgroup analyses and meta regression results.

	Number of models	Sen^1^		Spe^2^		PLR		NLR		DOR		AUC	Meta regression	
		Pooled (95%CI)	*I* ^*2*^	Pooled (95%CI)	*I* ^*2*^	Pooled (95%CI)	*I* ^*2*^	Pooled (95%CI)	*I* ^*2*^	Pooled (95%CI)	*I* ^*2*^		*P*	RDOR
Layers													0.4878	0.74
≤10	5	1.00 (1.00–1.00)	97.80%	0.99 (0.99–0.99)	60.20%	104.30 (70.94–153.36)	65.70%	0 (0.00–0.03)	98.60%	35609.58(4947.73–256287.76)	93.80%	0.9992		
10–20	28	0.91(0.92–0.91)	99.70%	0.97 (0.97–0.96)	99.80%	7.40 (5.04–10.86)	99.20%	0.10 (0.07–0.16)	99.60%	52.47 (33.55–82.06)	98.50%	0.9861		
20–50	11	0.94(0.93–0.95)	96.70%	0.95 (0.95–0.96)	97.80%	28.68 (10.64–77.28)	98.30%	0.04 (0.02–0.09)	96.40%	770.17 (163.74–3622.58)	97.90%	0.9927		
50–100	7	0.93(0.91–0.94)	85.40%	0.98 (0.97–0.98)	72.10%	36.07 (30.09–43.24)	50.80%	0.10 (0.06–0.16)	70.70%	366.71 (219.09–613.82)	59.70%	0.9935		
>100	4	0.95 (0.94–0.97)	64.80%	0.98 (0.97–0.99)	93.70%	76.90 (21.55–274.39)	90.70%	0.04 (0.03–0.08)	57.10%	1967.84 (464.67–8333.64)	84.40%	0.9914		
Type of AMD													0.1882	36.03
Single	2	1.00 (0.99–1.00)	0%	0.99 (0.98–1.00)	89.0%	122.44 (6.08–2463.89)	87.70%	0.00 (0.00–0.02)	0%	107120.15 (8471.87–1354450.49)	0%	NA^3^		
All	54	0.94 (0.94–0.94)	99.70%	0.97 (0.97–0.97)	99.70%	20.61 (14.59–29.12)	99.30%	0.06 (0.04–0.09)	99.70%	306.14 (187.38–500.18)	99.20%	0.9915		
Architectures													0.0004	0.19
ResNet	13	0.94 (0.93–0.95)	96.10%	0.96 (0.95–0.96)	97.60%	34.66 (13.66–87.94)	98.20%	0.04 (0.02–0.08)	96.00%	951.18 (232.03–3899.24)	97.70%	0.9941		
VGG	16	0.90 (0.90–0.89)	99.60%	0.91 (0.91–0.90)	98.50%	4.29 (3.25–5.67)	98.30%	0.19 (0.13–0.27)	99.50%	30.62 (21.91–42.79)	96.90%	0.8972		
Others	26	0.99 (0.99–0.99)	99.50%	0.99 (0.99–0.99)	99.40%	86.04 (33.97–217.95)	99.50%	0.01 (0.00–0.08)	99.70%	7354.18 (1231.62–43913.09)	99.40%	0.9987		
Types of Images													0.0002	0.12
OCT	36	0.94 (0.94–0.94)	99.80%	0.97 (0.97–0.97)	99.70%	52.33 (32.57–84.10)	99.50%	0.03 (0.02–0.04)	99.70%	2209.74 (1113.02–4387.10)	99.40%	0.9982		
FP	11	0.83 (0.81–0.84)	96.90%	0.96 (0.96–0.97)	98.20%	9.26 (3.74–22.92)	99.10%	0.18 (0.06–0.53)	99.10%	51.02 (13.37–194.69)	97.70%	0.9592		
OCT & FP	8	0.84 (0.83–0.85)	45.70%	0.88 (0.87–0.89)	87.00%	4.38 (3.11–6.17)	91.30%	0.18 (0.15–0.20)	30.00%	28.80 (20.38–40.71)	70.70%	0.9176		
OPTOS	1	1.00 (0.97–1.00)	0%	0.97 (0.94–0.99)	0	34.95 (20.45–59.73)	0%	0	0	9371.15 (523.79–167661.33)	0%	NA		

Sen: Sensitivity; Spe: Specificity; NA: not applicable.

## Discussion

This meta-analysis included 18 studies and 56 models aimed to investigate the performance of deep learning in detecting AMD. The results of the present study indicate a high accuracy in detecting AMD through CNN, but with high heterogeneity. The sources of heterogeneity were the types of AMD and layers of the network according to the meta-regression.

DL has been widely adopted in image recognition, speech recognition, and natural language processing, but is only beginning to impact healthcare, especially in ophthalmology [6]. DL is a subset of machine learning which has become possible with increasing computing power. Compared to traditional machine learning algorithms and shallow networks, current DL algorithms are characterized by large amounts of processable data, high computational power, and large network size [58, 59].

Fluorescein angiography, optical coherence tomography (OCT), optical coherence tomography angiography (OCTA), FP, fundus autofluorescence, and indocyanine green angiography are useful diagnostic tests in clinical practice to detect AMD [1], of which OCT and FP are the most commonly used. Plenty of public ophthalmic datasets are based on the above two types of images, which have facilitated the rapid development of artificial intelligence in ophthalmology, and will make telemedicine more convenient in the future. This meta-analysis reveals that DL detection through OCT, FP, and OPTOS ultra-widefield retinal images has a high accuracy in AMD diagnosing.

18 included studies were summarized in **Table 2**. All studies adopted CNN to conduct DL models. The non-saturating ReLU activation function was introduced in AlexNet to increase the training speed and the dropout method was used to minimize overfitting in the fully connected layers [60]. VGG has a deeper architecture, but cannot overcome the limitation of the vanishing gradient problem [61]. In the ResNet architecture, identity mapping is introduced to solve the vanishing gradient problem. ResNet can therefore be used to train deeper models [62]. DenseNets can alleviate the vanishing-gradient problem, strengthen feature propagation, encourage feature reuse, and substantially reduce the number of parameters [63]. Other studies mostly used a self-created CNN architectures with 7–20 layers. Duke, Mendeley, and Noor are the most used OCT databases. Most FP image datasets were built clinically. 10 studies [40, 41, 43, 46, 47, 52–55, 57] included more than 1000 images in their research. 4 studies [40, 43, 47, 57] included more than 100 thousand images. Only Matsuba et al [50] used OPTOS as the dataset, which is unique and pioneering among the 18 studies. Dong et al [43] and Gour et al [44] included 11 and 8 classes respectively, while other studies only contained 2–4 classes.

In this research, the type of AMD and the layers of the network were found to be the two essential factors that impact the accuracy of the diagnosis. However, the layers of the network are not positively correlated with diagnostic accuracy. Even though DOR and AUC are higher when the layers are less than 10, as layers of the network are more than 10, the diagnostic accuracy gradually grows as the number of layers increases. Cautiously, when the number of layers becomes too deep, overfitting may occur. Overfitting is a serious issue when training DL models, which may cause the trained models cannot be generalized in other data or datasets [64]. Predictably, deeper and more accurate networks will be placed in service soon. Meanwhile, different types of AMD may make the computation more difficult, but the prevailing datasets contain different types of AMD, which will make the trained models more generalized.

Although the meta-regression results did not show that the networks and types of images connected to the diagnostic accuracy, they are still significant. The DOR of ResNet showed superior than VGG, other variants cannot be assessed because they were only included in one study. That might be because ResNet with more layers was developed after VGG. ResNet belongs to deep residual networks with a higher amount of processable data [65]. ResNets can be trained easily without increasing the training error percentage, and are helpful in tackling the vanishing gradient problem using identity mapping [66]. Therefore, it is believed ResNet is an ideal architecture among the present variants of CNN. However, the influence due to the layers of the network impacts the results. This may be the reason the RDOR of networks in meta-regression is very low. As for the types of images, OCT images showed superior in detecting AMD. OCT images can reveal every layer of macular structures with more anatomical information than fundus images. Combined OCT images with fundus images had worse sensitivity, specificity, DOR, and AUC. We think it is because two images have more information. More information means more computation and the potential to be more accurate, which may require considering the layers of the network and the architecture of CNNs. Additionally, although only one study [50] reported the OPTOS ultra-widefield retinal images as self-dataset, the pooled sensitivity, specificity, and DOR were all highest in the four subgroups.

The detailed limitations for each study were summarized in **Table 3**. Generally, 8 studies [42, 47, 50–54, 56] did not study other retinal diseases. 3 studies [45, 49, 55] only contained one other diseases. 2 studies [48, 56] had small datasets with no more than 100 images. Bhatia et al [41] ignored bad quality pictures that may cause a generalization issue. Celebi et al [42] ignored bad quality pictures and patients who had other retinal diseases. Although Dong et al [43] established a database with 11 classes, the number of retinitis pigmentosa images is small. Gour et al [44] contained 8 types of diseases, but with a small dataset of 331 FP images. Kadry et al [46] used 4 CNN variants for classification, but the definition of non-AMD is not clear. Lee et al [47] included only images from patients who met the study criteria, and the neural network was only trained on these images. Meanwhile, the model was trained using images from a single academic center, and the external generalizability is unknown. Mathews et al [49] used drusen macular degeneration for AMD diagnosis. Matsuba et al [50] used OPTOS images, but it is difficult to acquire precise images using OPTOS when the transmission of light into the eye is impaired by an intermediate translucent zone. At the same time, most AMD patients accept treatment which may cause diagnostic errors. Motozawa et al [51] excluded low quality images and patients who had other concomitant diseases. Zapata et al did not report a clear number of OCT or FP images.

This meta-analysis and the included studies have several limitations. First, some variants of CNN including CapsNet, AlexNet, and DenseNet only existed once, and some studies used self-created CNN architectures. Therefore, the subgroup analysis of networks is not accurate. Second, we tried to establish more subgroups or to find more possible covariates such as hardware, network, and hyperparameters. However, these potential factors were not mentioned in many studies. Third, we concentrated more on diagnostic accuracy, but as DL develops, AMD diagnostics has become more diverse, more plentiful, and more useful in lesion segmentation and efficacy prediction, which will be highly considered for further research. Forth, the Duke and some self-built datasets have a small number of images for training.

### Future challenges and direction

DL is still in the early stages of development in AMD diagnosis, but in the foreseeable future, widespread use could play a significant role in fundus applications, screening, telemedicine, reducing the workload of physicians, etc.

The purpose of DL algorithms for diagnosing AMD is to achieve an automated diagnosis of many kinds of fundus diseases. However, no matter in public databases or self-built databases, only several diseases were chosen for classification which is difficult for widespread use clinically. Establishing a database which covers heterogeneous and large image sets is still a serious challenge. Meanwhile, the DL algorithms concentrate more on images only, but the images are not the only data obtained clinically. Ideally, multimodal data containing clinical data, FP, and OCT, etc. may increase the diagnostic accuracy. At the same time, traditional fundus datasets mostly consist of FP and OCT images. However, with diagnostic tests developing, more new methods and technologies such as OPTOS ultra-widefield retinal images, OCTA, FFA, ICGA, etc. will be added as public or self-built datasets in future AMD detection. Finally, as the equipment evolves, the image quality of FP, OCT, OCTA etc. improves. More high definition images will increase the diagnostic accuracy.

## Conclusions

CNNs are mostly adopted deep learning algorithms in AMD detection. All included DL algorithms adopted CNNs. CNNs, especially ResNets, are effective in detecting AMD with high diagnostic accuracy. The types of AMD and the layers of the network are the two essential factors that impact the model training process. Proper layers of the network will make the model more reliable. More datasets established by new diagnostic methods such as ultra-widefield retinal images, FFA, and ICGA will be used to train DL models in the future, which will be helpful in fundus application screening, long-range medical treatment, and reducing the workload of physicians.

## Supporting information

S1 Fig The original forest plots. (PDF)

S1 Table PRISMA checklist. (DOC)

S2 Table PRISMA DTA for abstracts checklist table. (DOC)

S1 File Search strategy. (DOCX)
