# Supplementary material for: Deep learning for detection of age-related macular degeneration: A systematic review and meta-analysis of diagnostic test accuracy studies
Source: PLoS One. 2023 Apr 6;18(4):e0284060. doi: 10.1371/journal.pone.0284060 (PMC10079062; doi:10.1371/journal.pone.0284060)

Figure 1S section A: The pooled results

Figure 1S section A (a): The pooled results of PLR

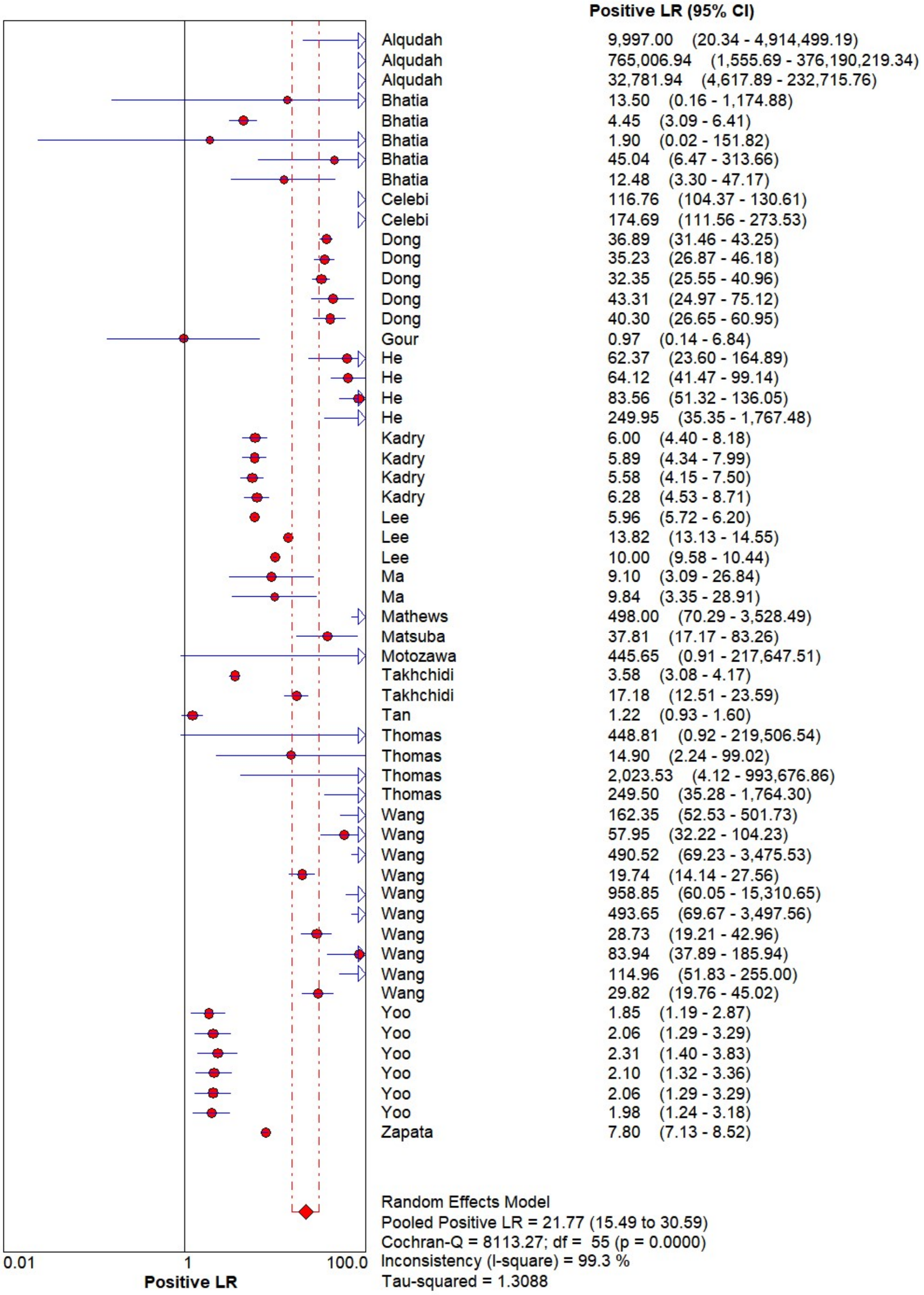

Figure 1S section A (b): The pooled results of NLR

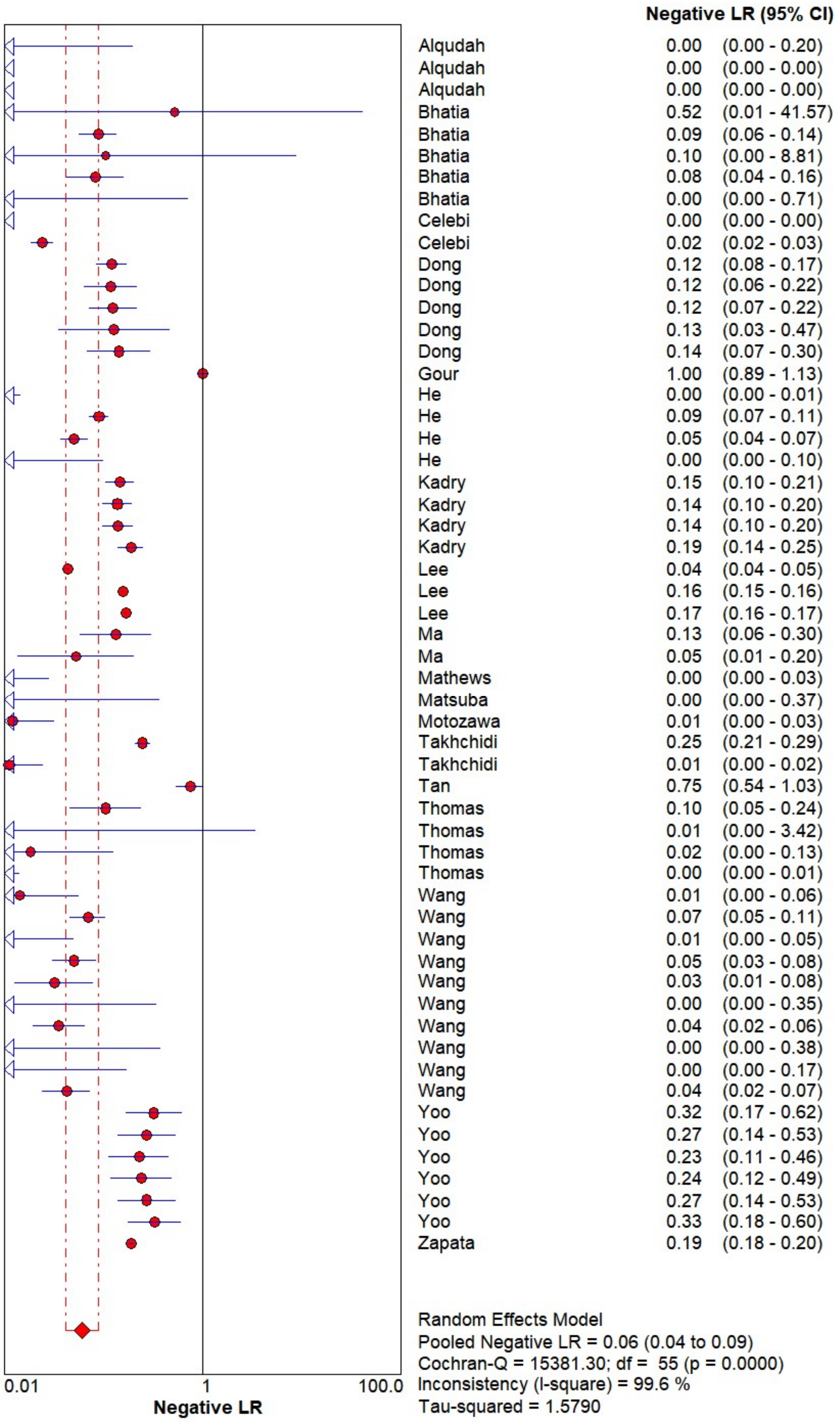

Figure 1S section A (c): The pooled results of DOR

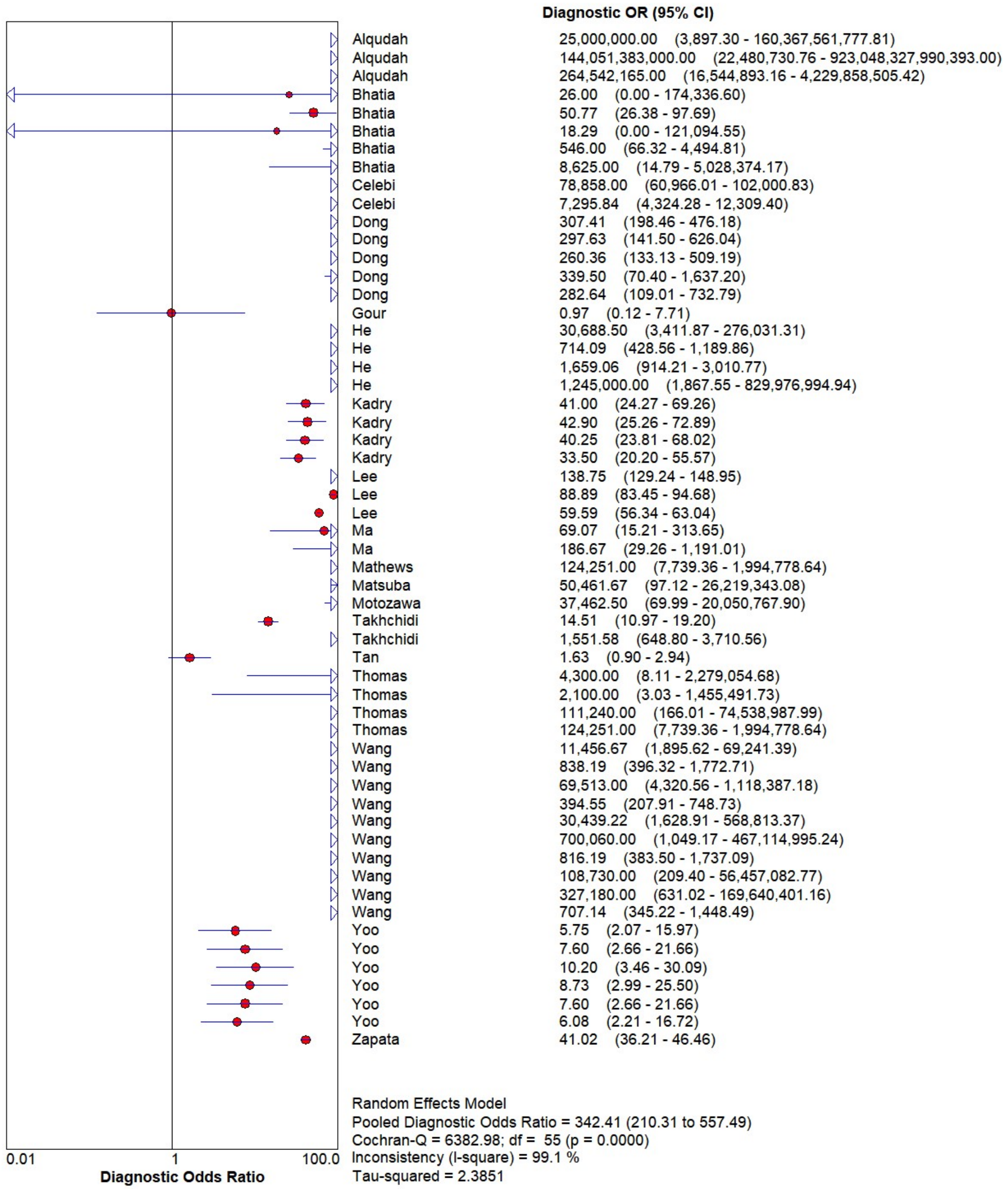

Figure 1S section B: The subgroup analysis of types of AMD

Figure 1S section B (a): The pooled results of sensitivity in subgroup of all types of AMD

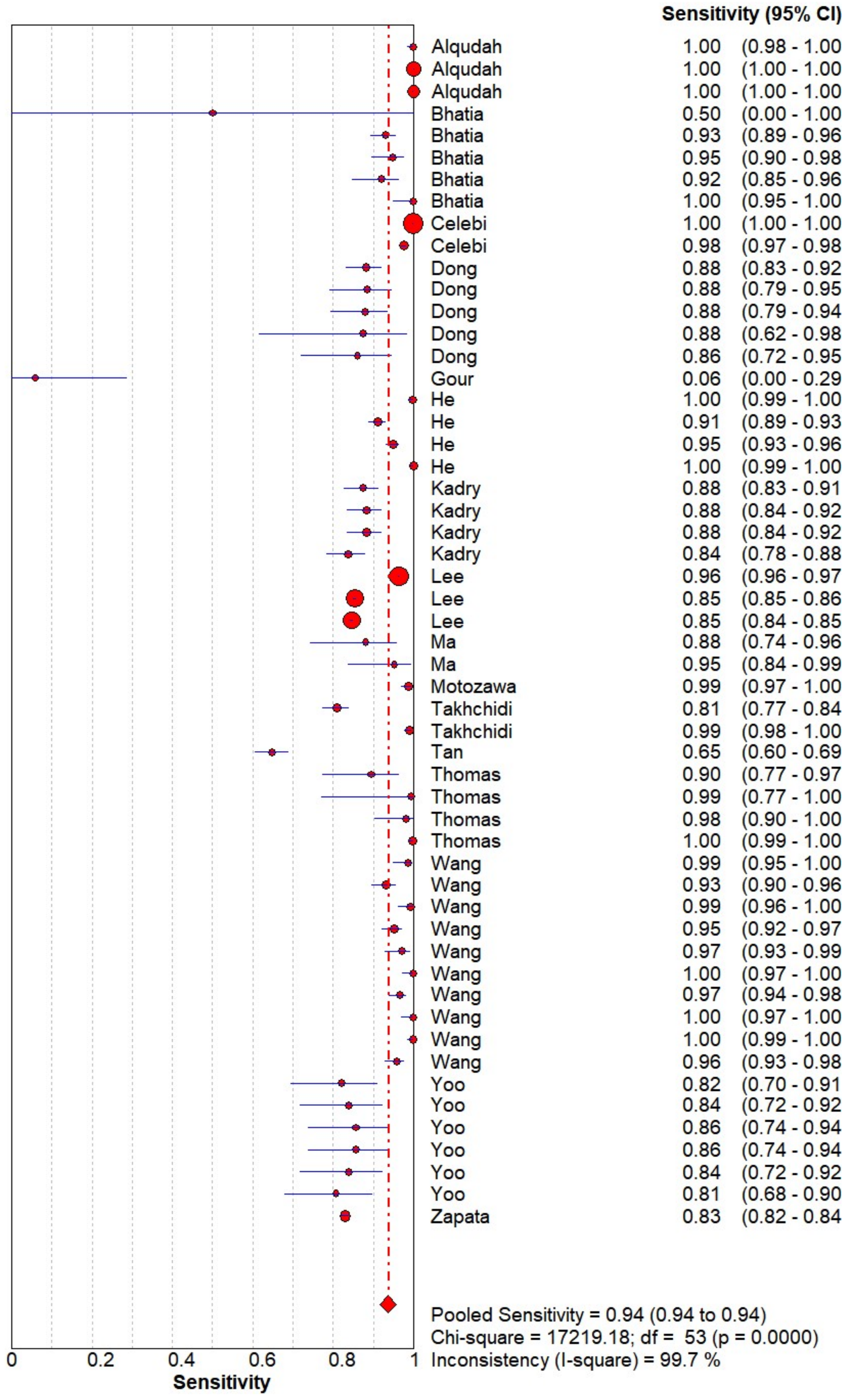

Figure 1S section B (b): The pooled results of specificity in subgroup of all types of AMD

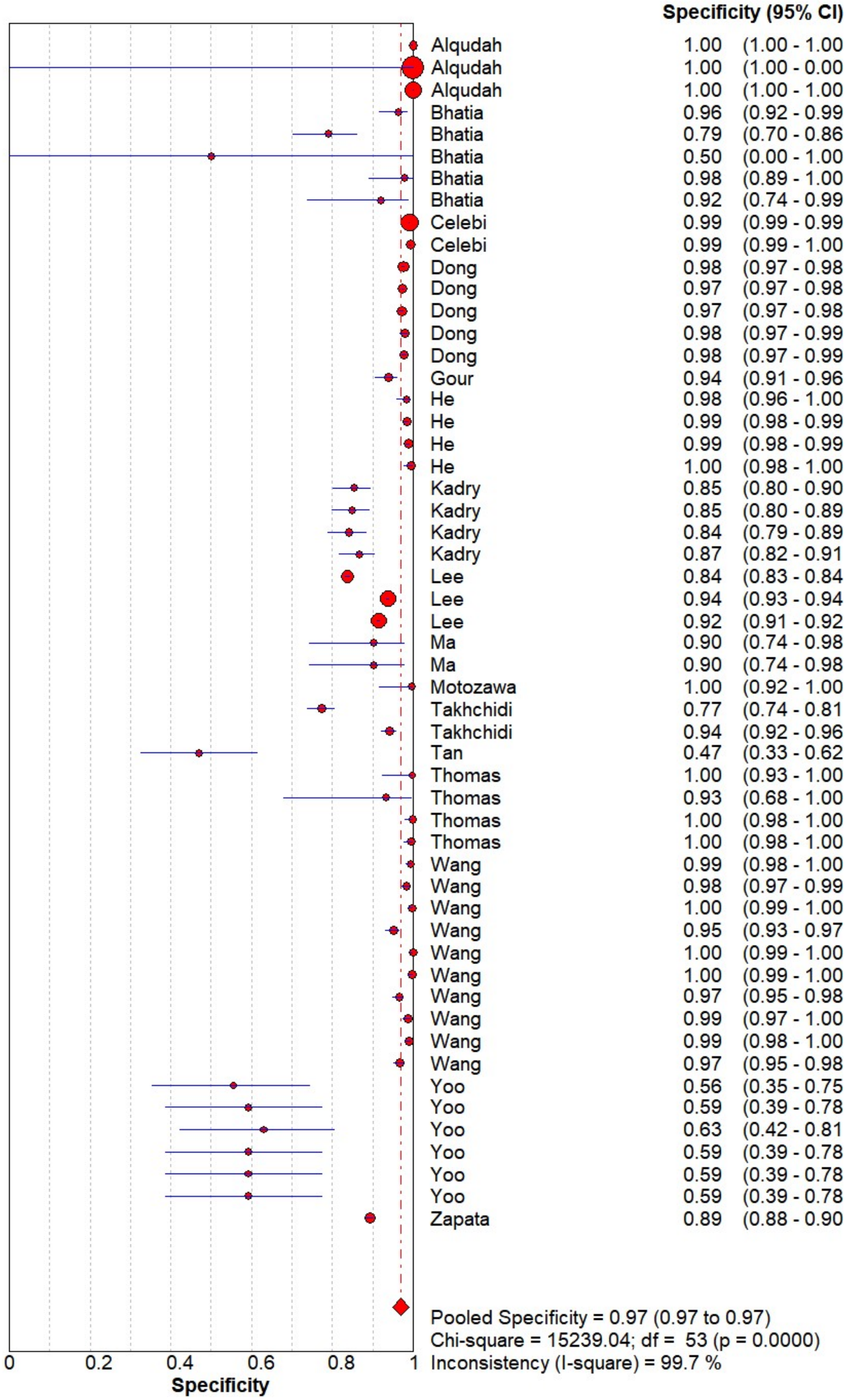

Figure 1S section B (c): The pooled results of sensitivity in subgroup of single types of AMD

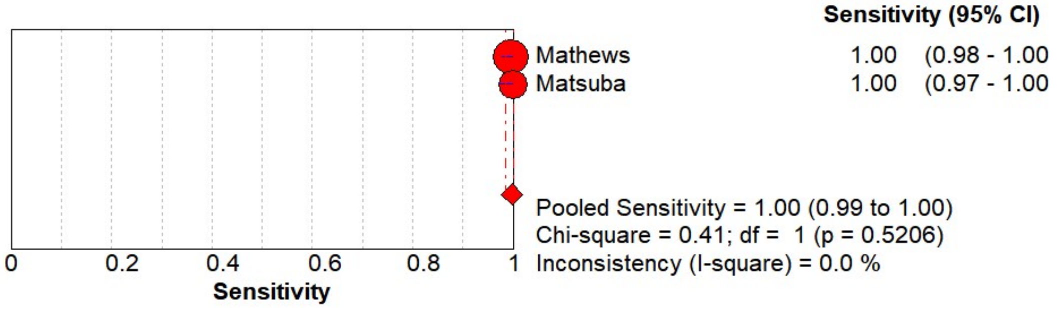

Figure 1S section B (d): The pooled results of specificity in subgroup of single types of AMD

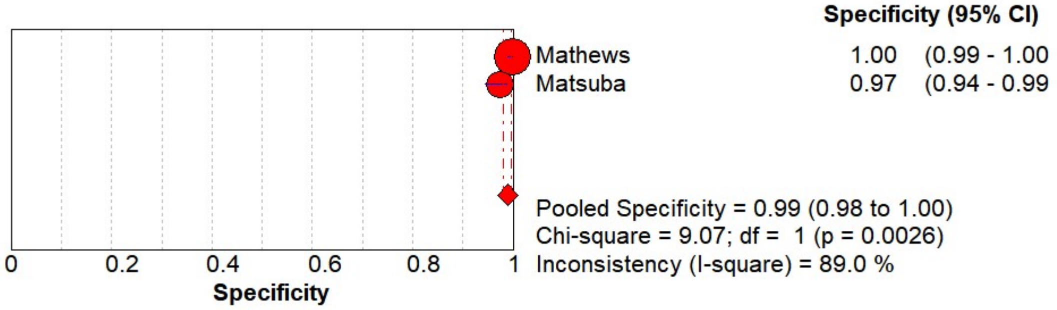

Figure 1S section C: The subgroup analysis of layers of the network

Figure 1S section C (a): The pooled results of sensitivity in subgroup of ≤10 layers

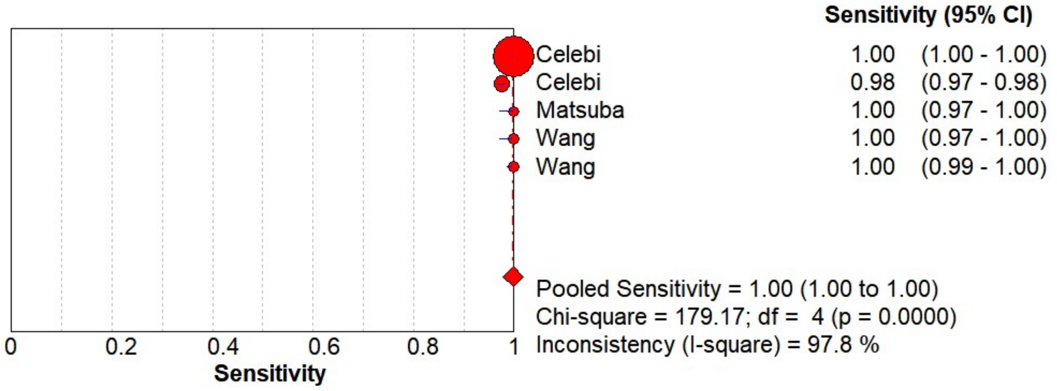

Figure 1S section C (b): The pooled results of specificity in subgroup of≤10 layers

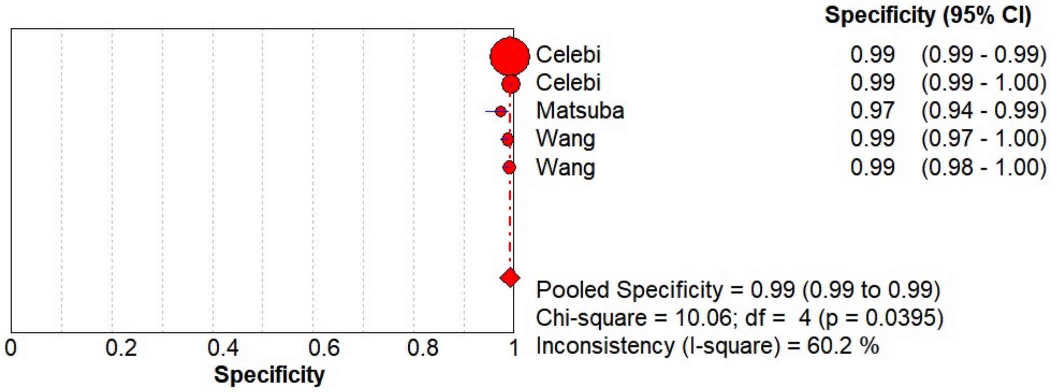

Figure 1S section C (c): The pooled results of sensitivity in subgroup of 10-20 layers

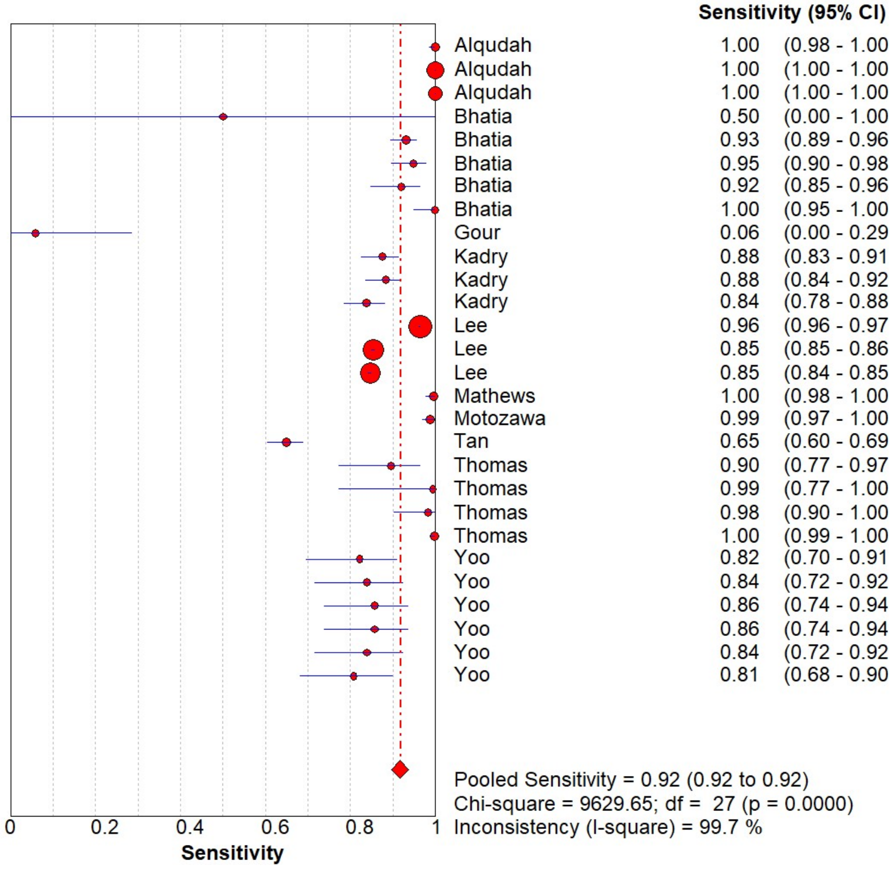

Figure 1S section C (d): The pooled results of specificity in subgroup of 10-20 layers

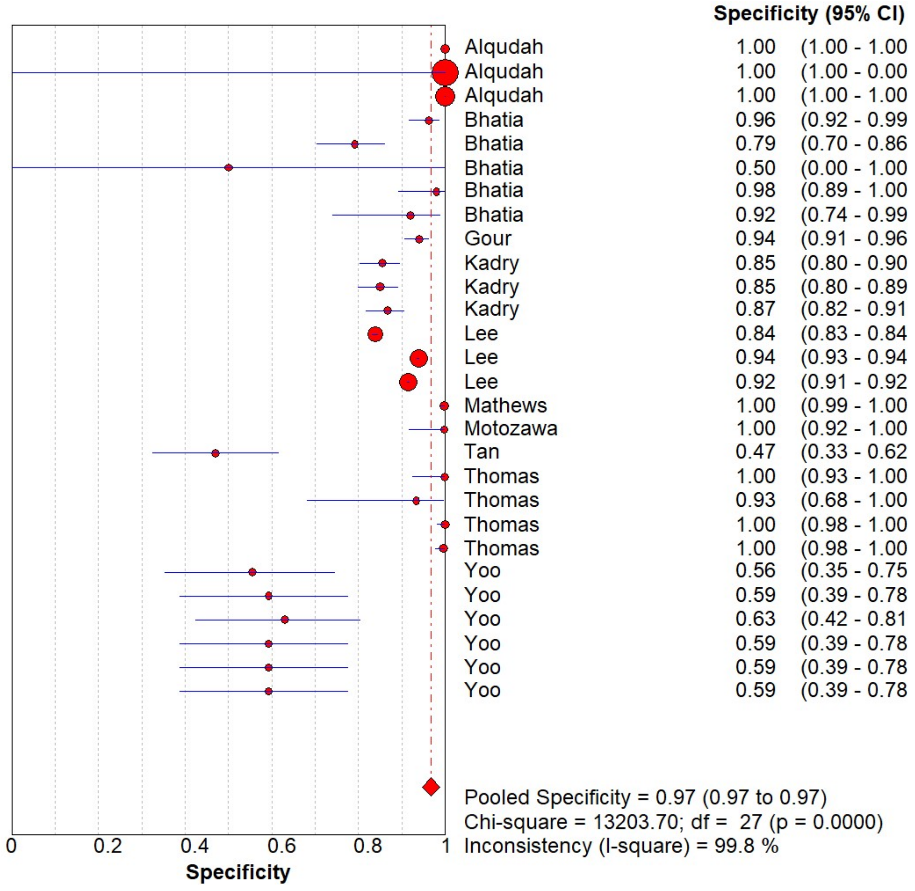

Figure 1S section C (e): The pooled results of sensitivity in subgroup of 20-50 layers

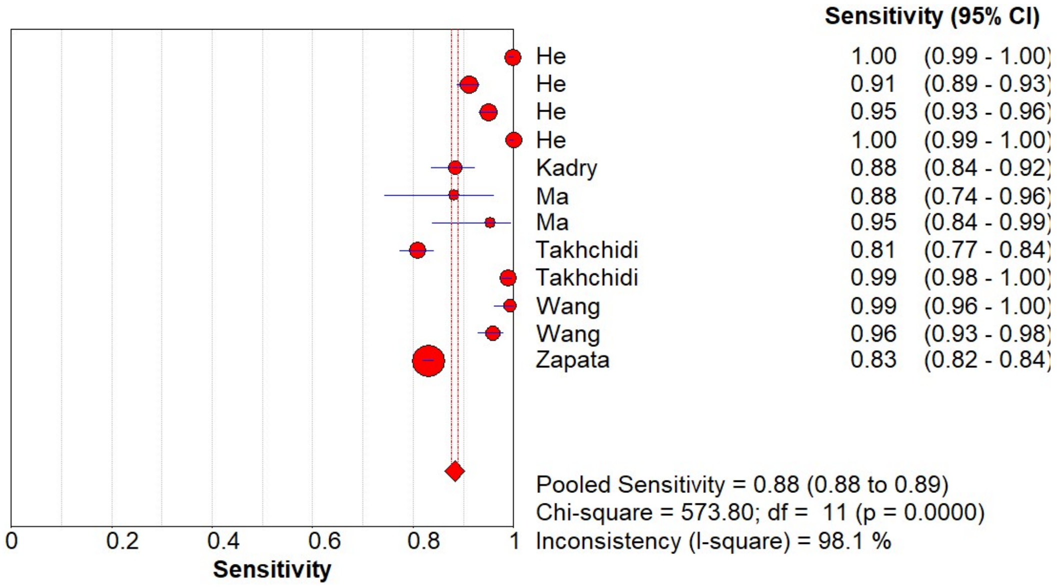

Figure 1S section C (f): The pooled results of specificity in subgroup of 20-50 layers

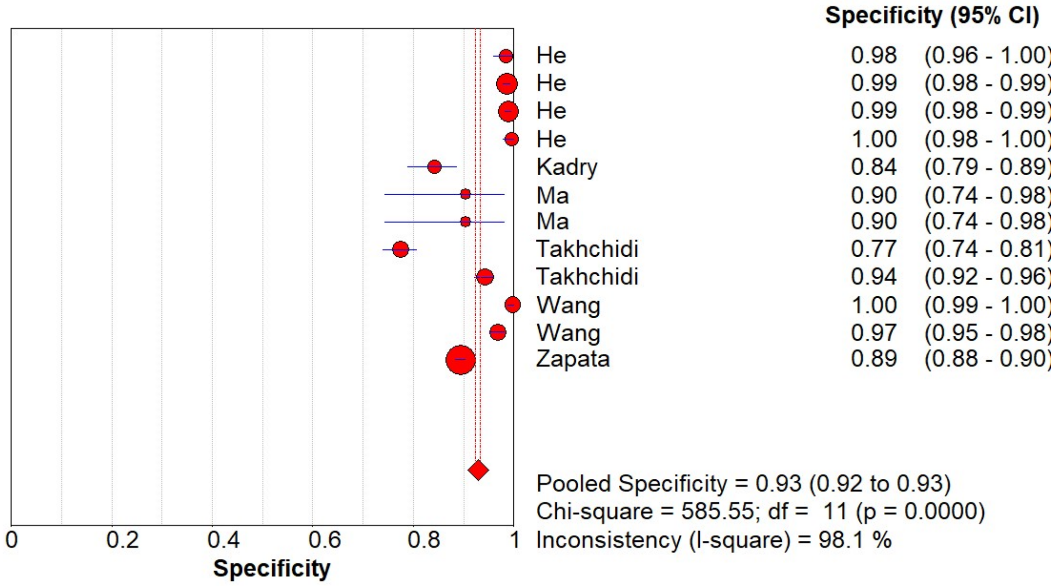

Figure 1S section C (g): The pooled results of sensitivity in subgroup of 50-100 layers

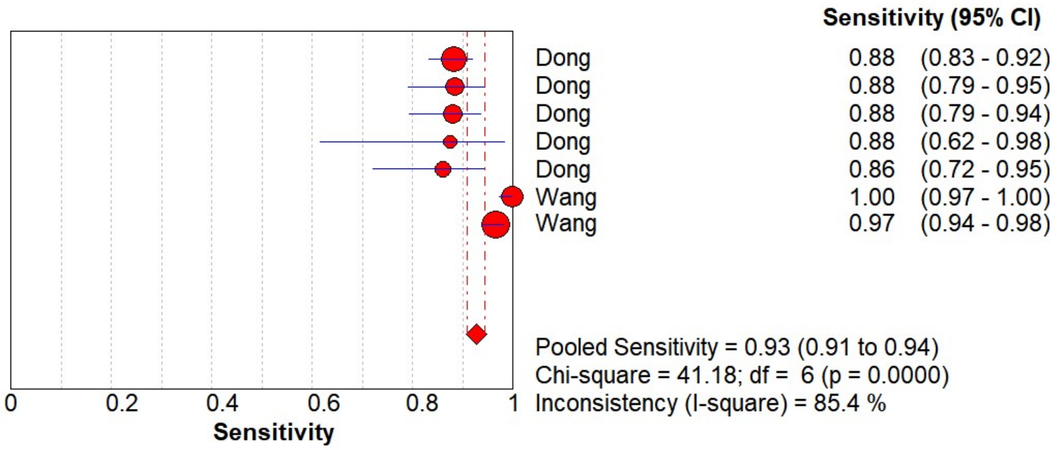

Figure 1S section C (h): The pooled results of specificity in subgroup of 50-100 layers

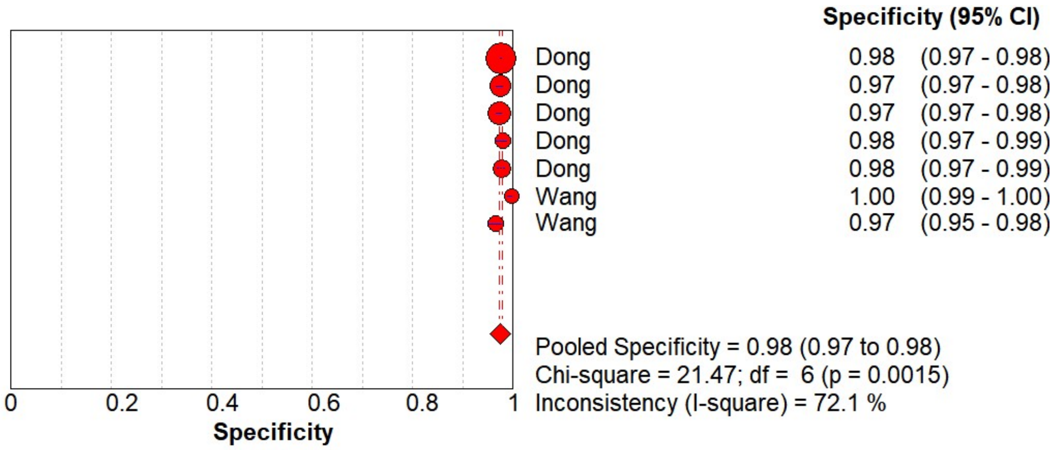

Figure 1S section C (i): The pooled results of sensitivity in subgroup of≥100 layers

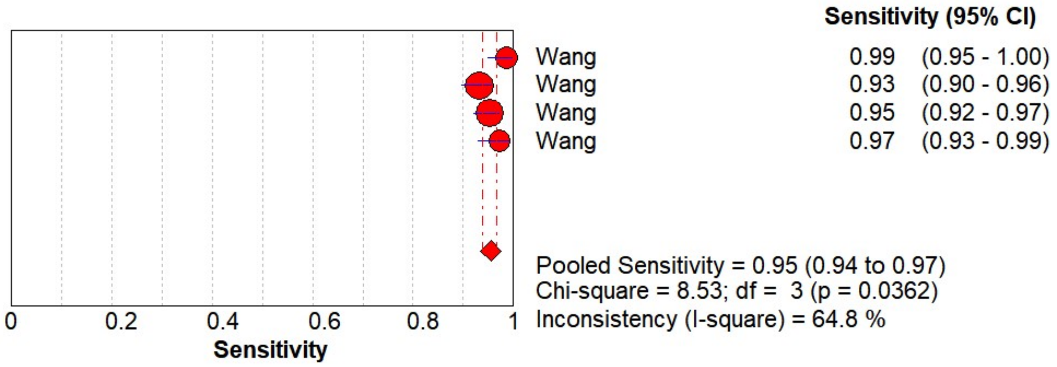

Figure 1S section C (j): The pooled results of specificity in subgroup of≥100 layers

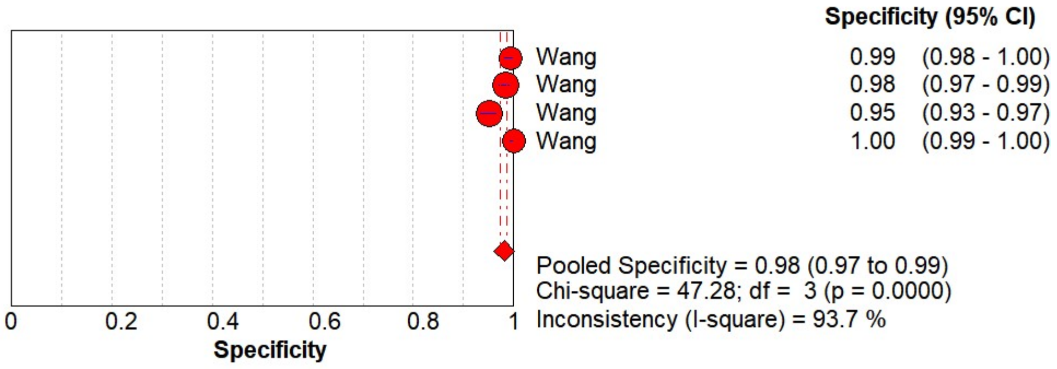

Figure 1S section D: The subgroup analysis of types of variants of CNN

Figure 1S section D (a): The pooled results of sensitivity in subgroup of ResNet

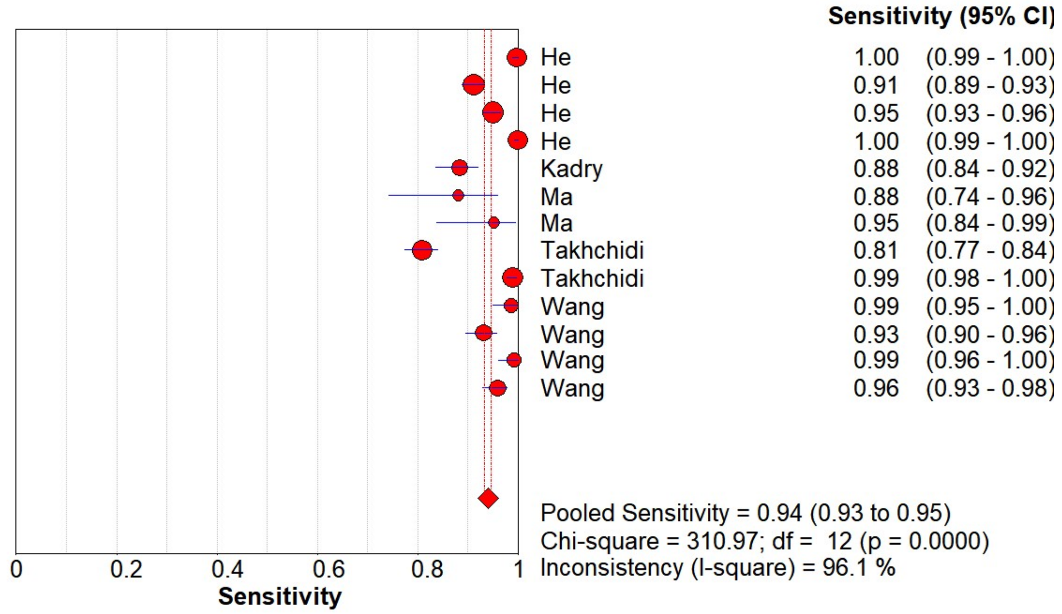

Figure 1S section D (b): The pooled results of specificity in subgroup of ResNet

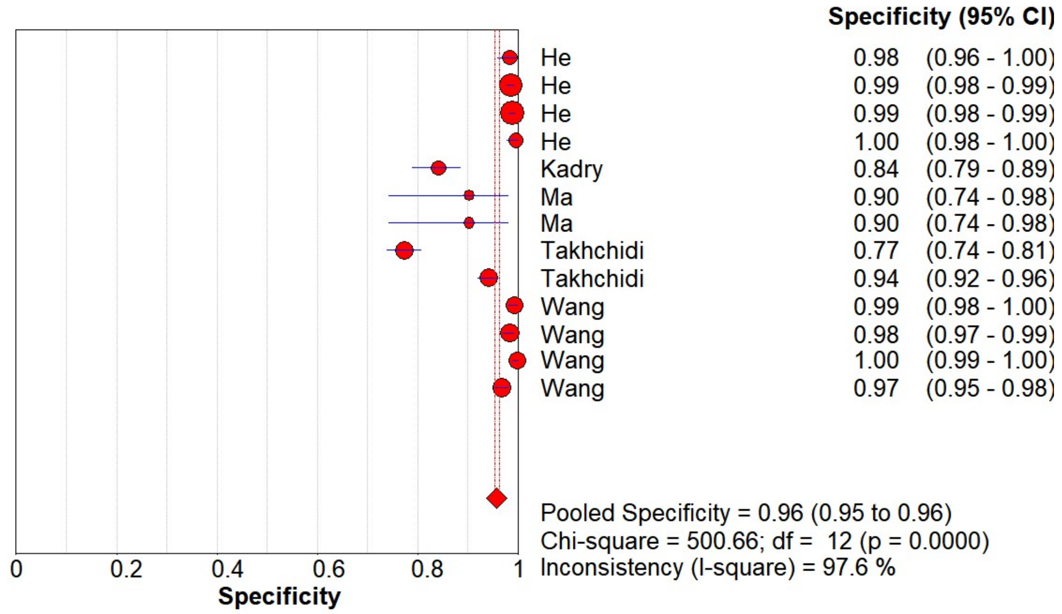

Figure 1S section D (c): The pooled results of sensitivity in subgroup of VGG

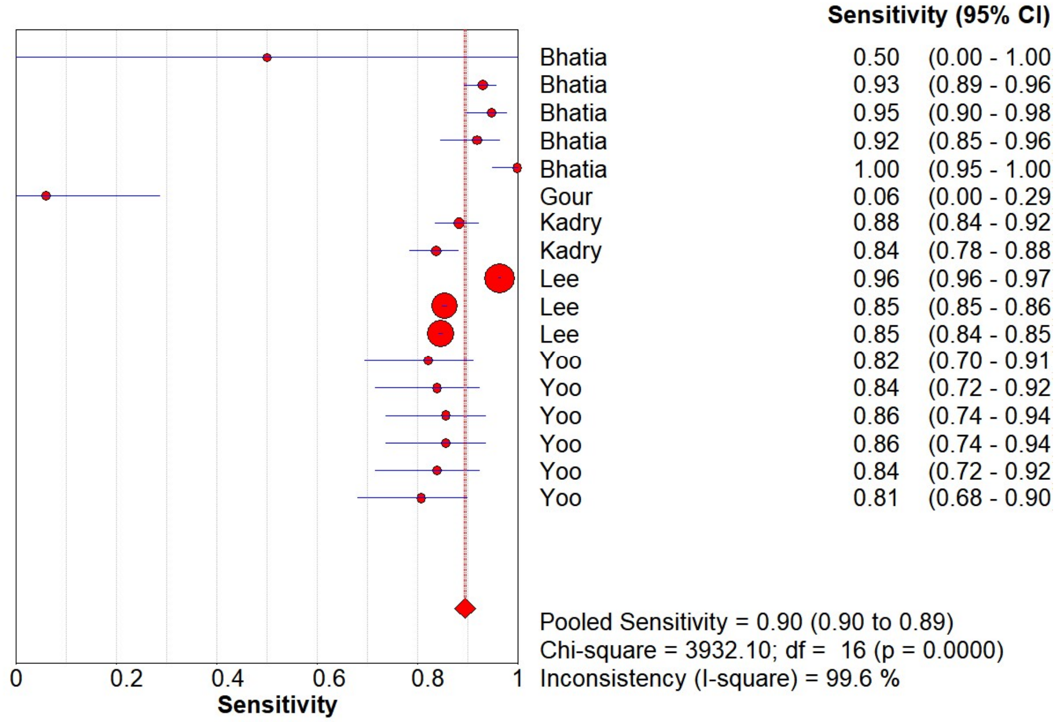

Figure 1S section D (d): The pooled results of specificity in subgroup of VGG

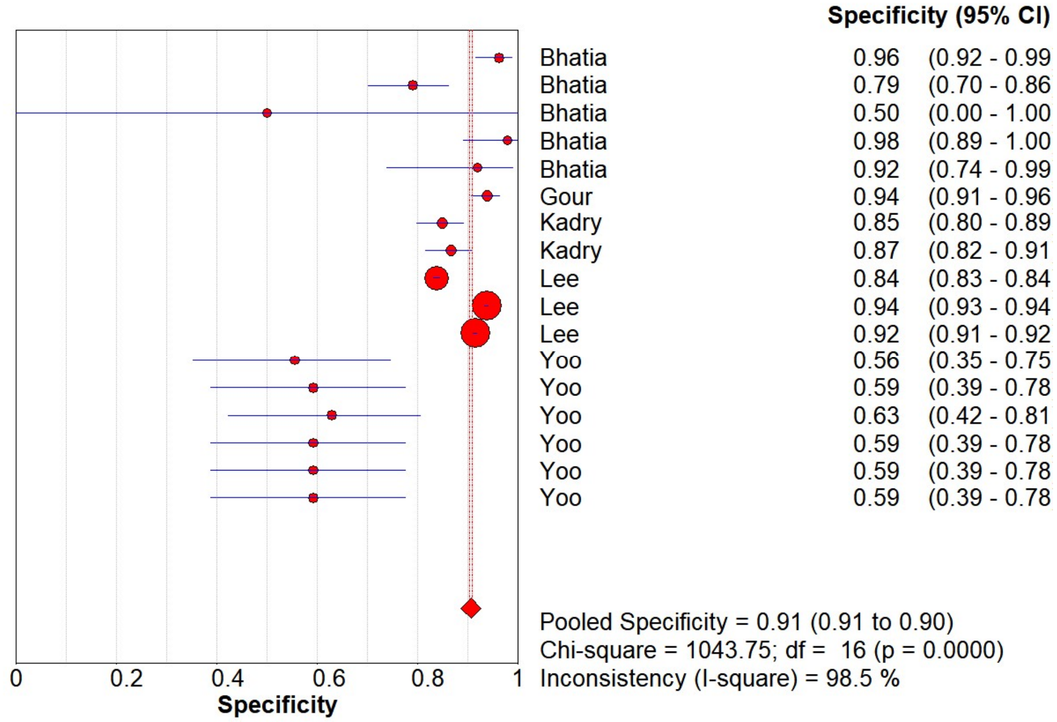

Figure 1S section D (e): The pooled results of sensitivity in subgroup of other networks

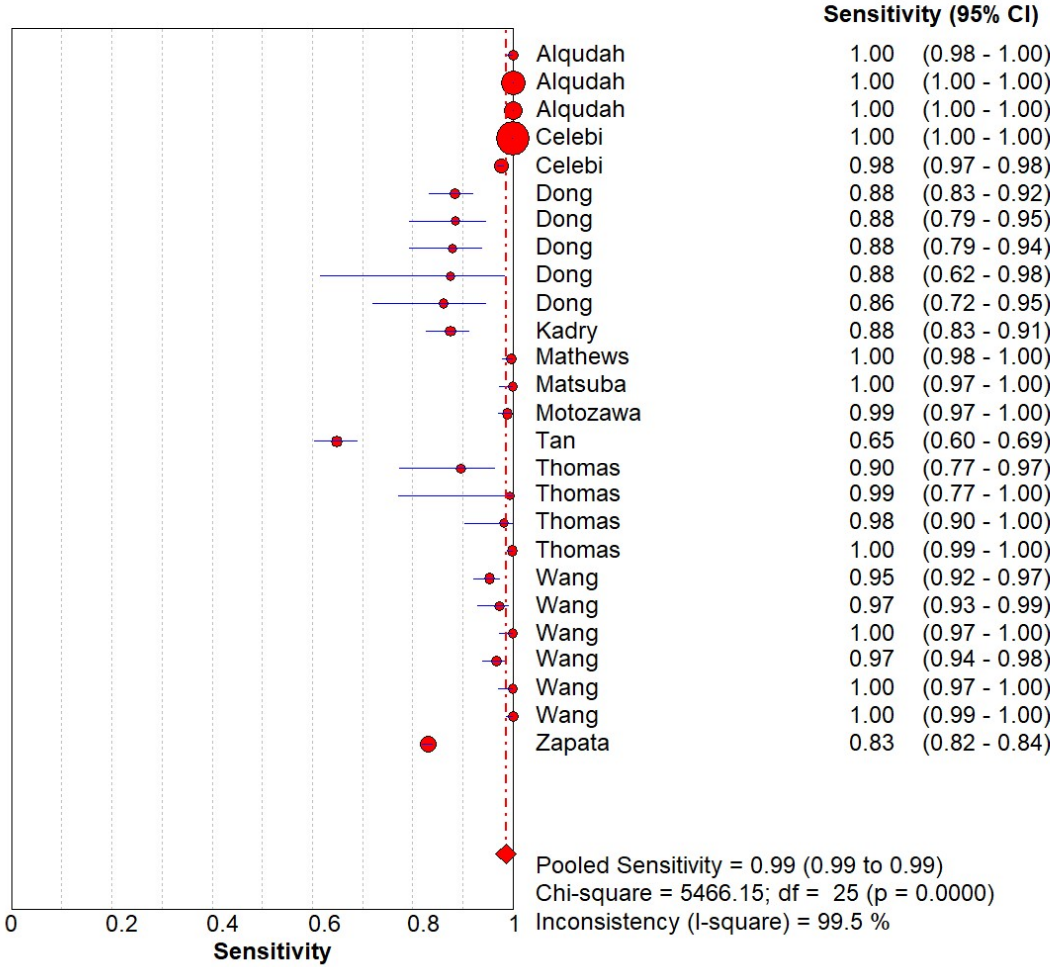

Figure 1S section D (f): The pooled results of specificity in subgroup of other networks

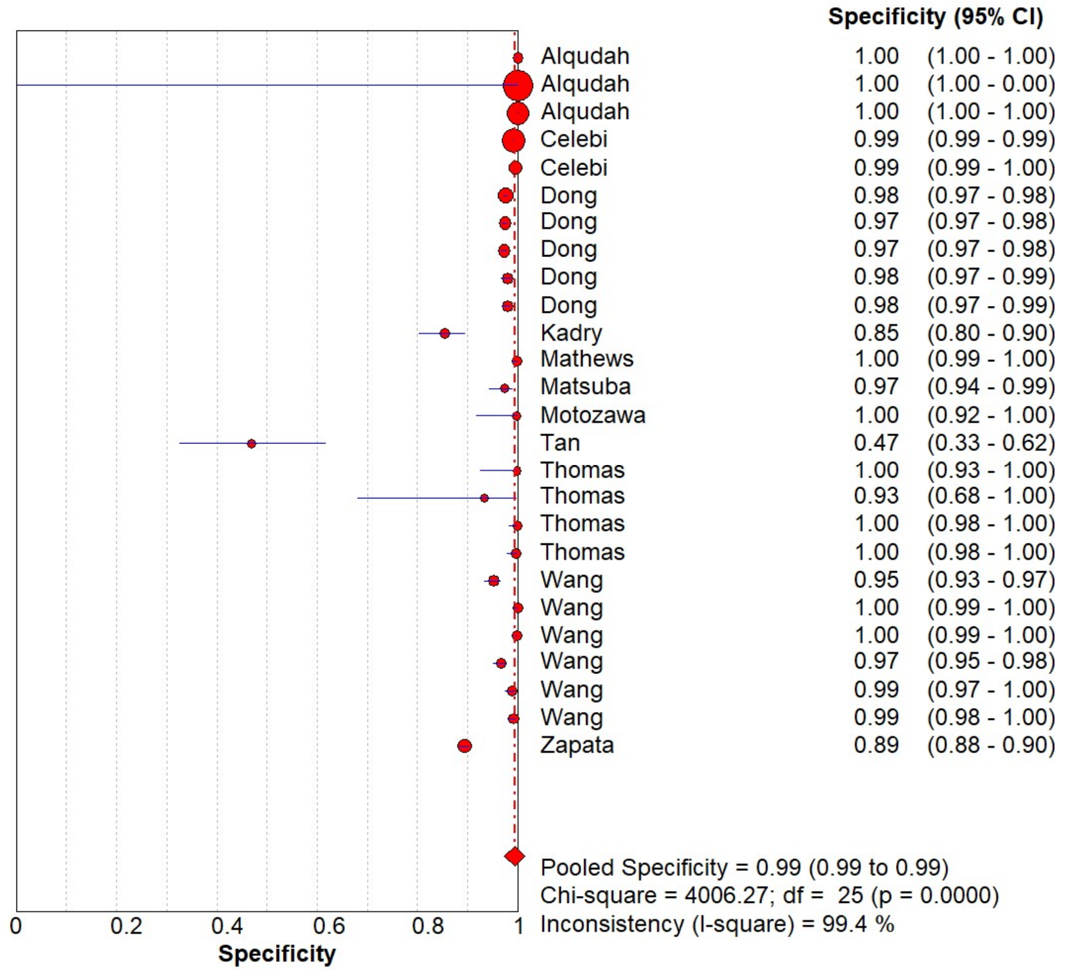

Figure 1S section E: The subgroup analysis of types of images

Figure 1S section E (a): The pooled results of sensitivity in subgroup of OCT

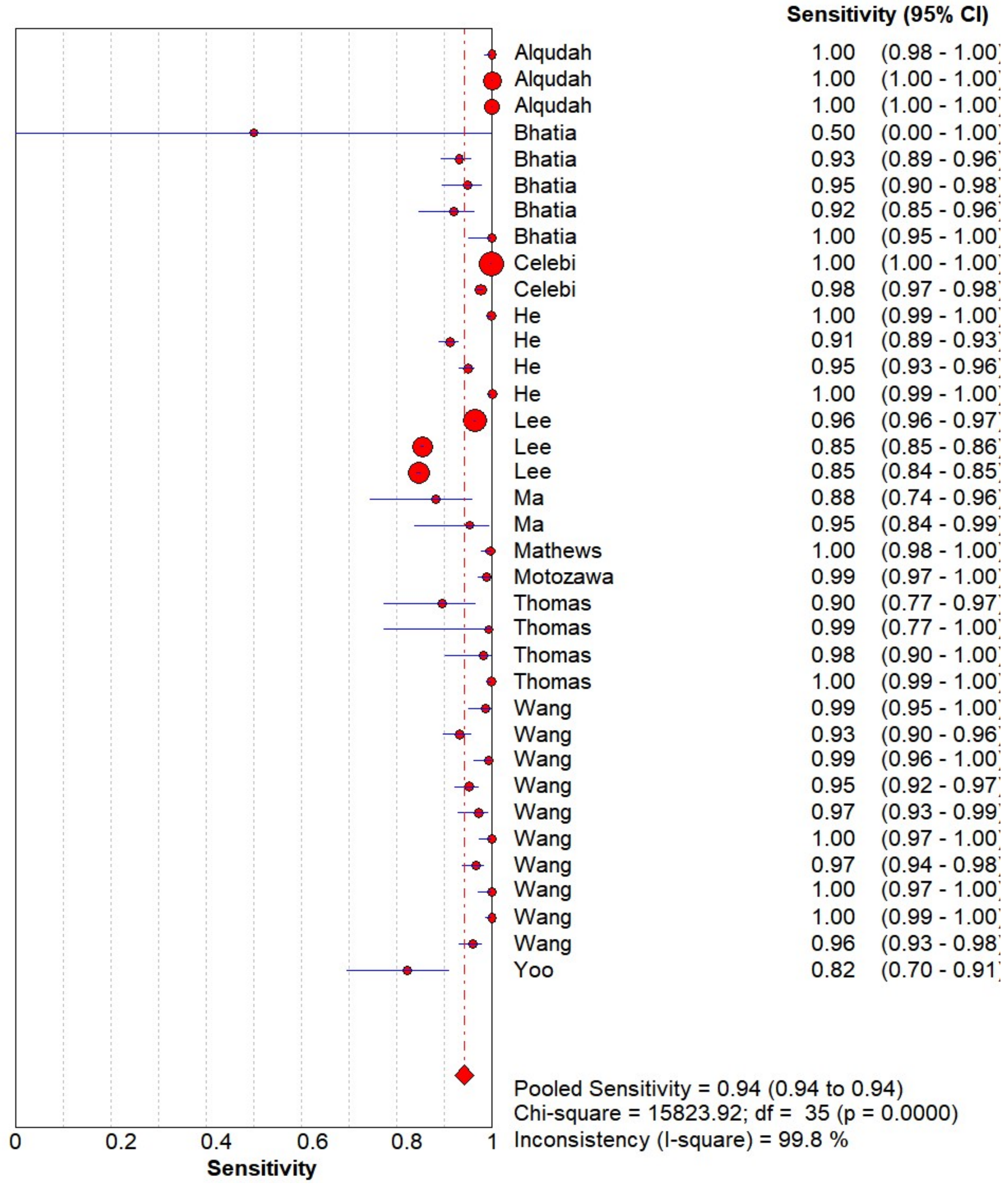

Figure 1S section E (b): The pooled results of specificity in subgroup of OCT

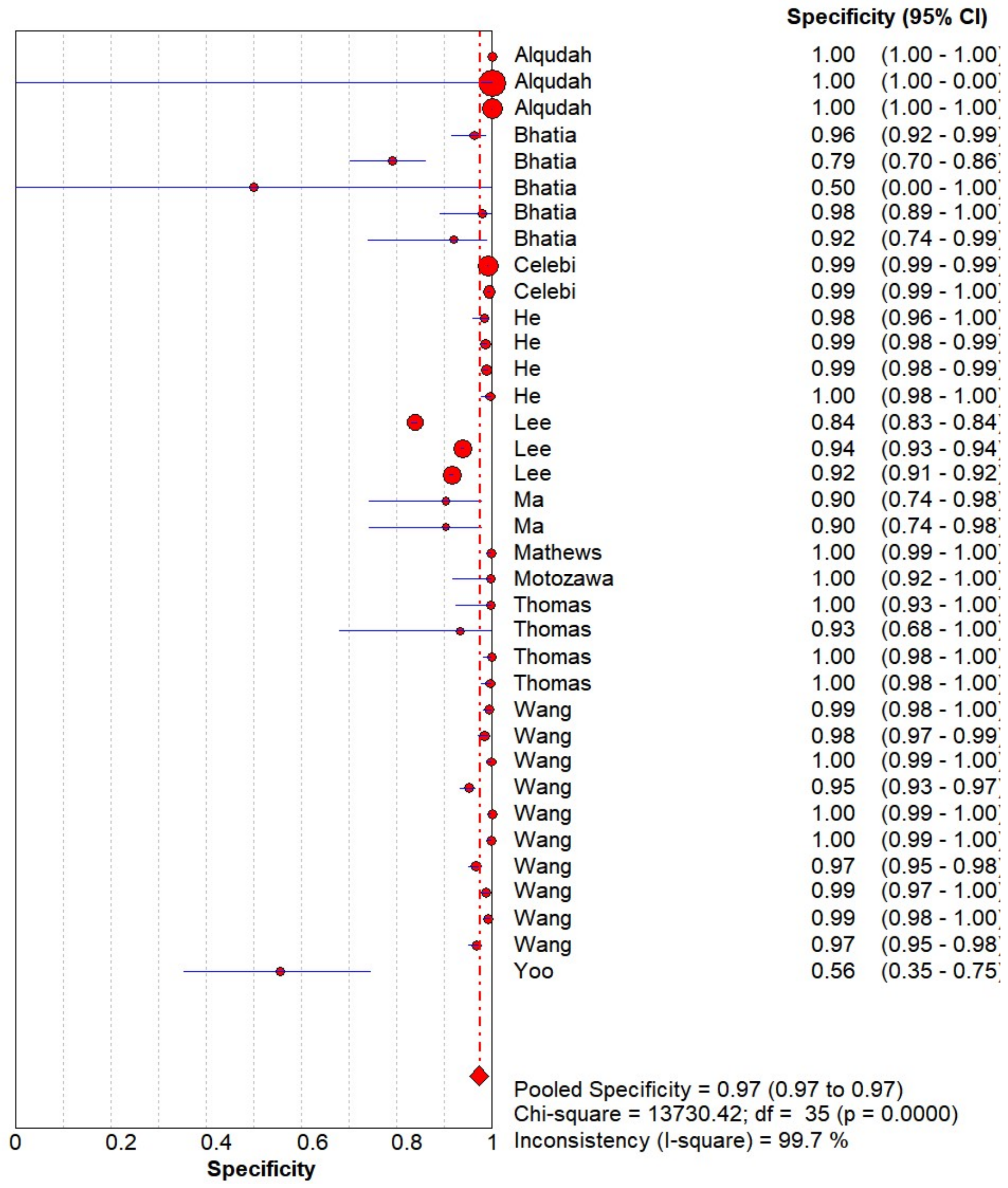

Figure 1S section E (c): The pooled results of sensitivity in subgroup of fundus photography

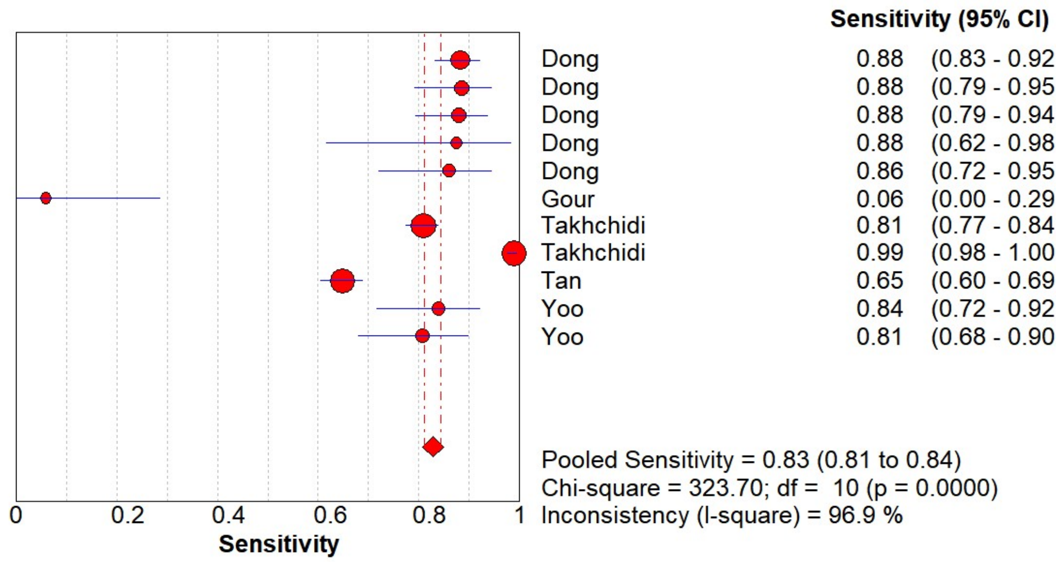

Figure 1S section E (d): The pooled results of specificity in subgroup of fundus photography

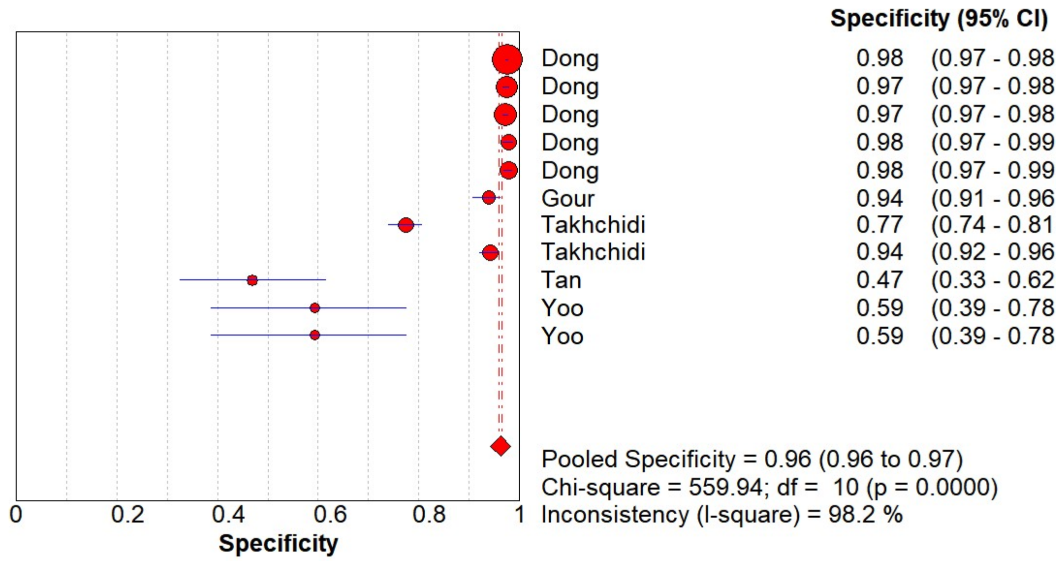

Figure 1S section E (e): The pooled results of sensitivity in subgroup of fundus photography combined with OCT

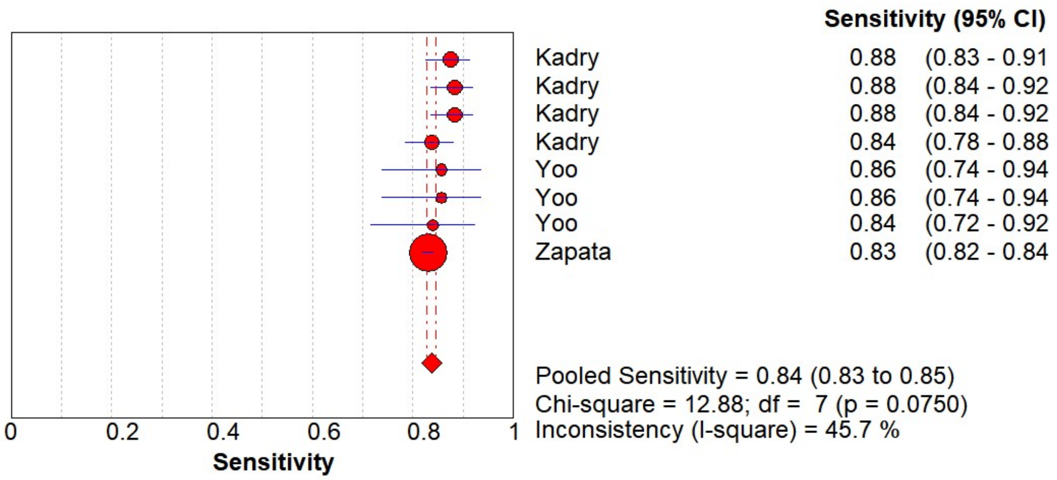

Figure 1S section E (f): The pooled results of specificity in subgroup of fundus photography combined with OCT

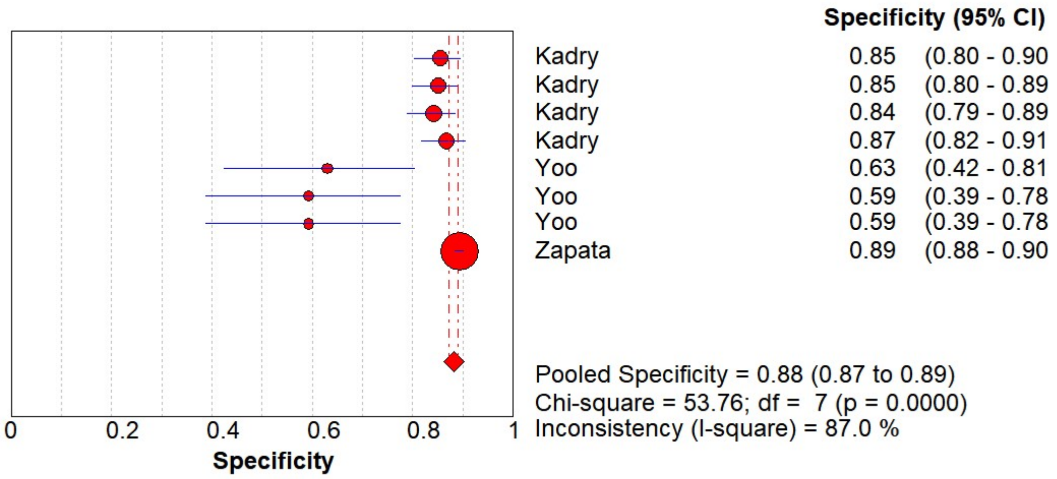

Supplement: S1 Fig — (PDF) [file pone.0284060.s001.pdf]
