# Supplementary material for: Deep learning for detection of age-related macular degeneration: A systematic review and meta-analysis of diagnostic test accuracy studies
Source: PLoS One. 2023 Apr 6;18(4):e0284060. doi: 10.1371/journal.pone.0284060 (PMC10079062; doi:10.1371/journal.pone.0284060)
Supplement: S2 Table — (DOC) [file pone.0284060.s003.doc]

| **Section/topic** | **#** | **PRISMA-DTA for Abstracts Checklist item** | **Reported on page #** |
| --- | --- | --- | --- |
| **TITLE and PURPOSE** | | |  |
| Title | 1 | Identify the report as a systematic review (+/- meta-analysis) of diagnostic test accuracy (DTA) studies. | 1 |
| Objectives | 2 | Indicate the research question, including components such as participants, index test, and target conditions. | 2 |
| **METHODS** | | |  |
| Eligibility criteria | 3 | Include study characteristics used as criteria for eligibility. | 2 |
| Information sources | 4 | List the key databases searched and the search dates. | 2 |
| Risk of bias & applicability | 5 | Indicate the methods of assessing risk of bias and applicability. | 2 |
| Synthesis of results | A1 | Indicate the methods for the data synthesis. | 2 |
| **RESULTS** | | |  |
| Included studies | 6 | Indicate the number and type of included studies and the participants and relevant characteristics of the studies (including the reference standard). | 2 |
| Synthesis of results | 7 | Include the results for the analysis of diagnostic accuracy, preferably indicating the number of studies and participants. Describe test accuracy including variability; if meta-analysis was done, include summary results and confidence intervals. | 2 |
| **DISCUSSION** | | |  |
| Strengths and limitations | 9 | Provide a brief summary of the strengths and limitations of the evidence | 2 |
| Interpretation | 10 | Provide a general interpretation of the results and the important implications. | 2 |
| **OTHER** | | |  |
| Funding | 11 | Indicate the primary source of funding for the review. | Cover Letter |
| Registration | 12 | Provide the registration number and the registry name | 6 |

*Adapted From:*  McInnes MDF, Moher D, Thombs BD, McGrath TA, Bossuyt PM, The PRISMA-DTA Group (2018). Preferred Reporting Items for a Systematic Review and Meta-analysis of Diagnostic Test Accuracy Studies: The PRISMA-DTA Statement. JAMA. 2018 Jan 23;319(4):388-396. doi: 10.1001/jama.2017.19163.

For more information, visit: **www.prisma-statement.org**.

Page 1 of 1
