# Supplementary material for: Deep learning for detection of age-related macular degeneration: A systematic review and meta-analysis of diagnostic test accuracy studies
Source: PLoS One. 2023 Apr 6;18(4):e0284060. doi: 10.1371/journal.pone.0284060 (PMC10079062; doi:10.1371/journal.pone.0284060)
Supplement: S1 File — (DOCX) [file pone.0284060.s004.docx]

**(a) PubMed search history**

| Search number | Query | Filters | Search Details | Results | Time |
| --- | --- | --- | --- | --- | --- |
| 1 | ((((((((((((((((((((Degeneration, Macular[Title/Abstract]) OR (Macular Degenerations[Title/Abstract])) OR (Maculopathy[Title/Abstract])) OR (Maculopathies[Title/Abstract])) OR (Macular Dystrophy[Title/Abstract])) OR (Dystrophy, Macular[Title/Abstract])) OR (Macular Dystrophies[Title/Abstract])) OR (Age-Related Macular Degeneration[Title/Abstract])) OR (Age Related Macular Degeneration[Title/Abstract])) OR (Age-Related Macular Degenerations[Title/Abstract])) OR (Macular Degeneration, Age-Related[Title/Abstract])) OR (Macular Degeneration, Age Related[Title/Abstract])) OR (Maculopathies, Age-Related[Title/Abstract])) OR (Maculopathy, Age-Related[Title/Abstract])) OR (Maculopathy, Age Related[Title/Abstract])) OR (Age-Related Maculopathies[Title/Abstract])) OR (Age Related Maculopathies[Title/Abstract])) OR (Age-Related Maculopathy[Title/Abstract])) OR (Age Related Maculopathy[Title/Abstract])) OR ("Macular Degeneration"[Mesh])) AND ((((Learning, Deep[Title/Abstract]) OR (·Hierarchical Learning[Title/Abstract])) OR (Learning, Hierarchical[Title/Abstract])) OR ("Deep Learning"[Mesh])) |  | ("degeneration macular"[Title/Abstract] OR "macular degenerations"[Title/Abstract] OR "Maculopathy"[Title/Abstract] OR "Maculopathies"[Title/Abstract] OR "macular dystrophy"[Title/Abstract] OR "dystrophy macular"[Title/Abstract] OR "macular dystrophies"[Title/Abstract] OR "age related macular degeneration"[Title/Abstract] OR "age related macular degeneration"[Title/Abstract] OR "age related macular degenerations"[Title/Abstract] OR "macular degeneration age related"[Title/Abstract] OR "macular degeneration age related"[Title/Abstract] OR "maculopathies age related"[Title/Abstract] OR "maculopathy age related"[Title/Abstract] OR "maculopathy age related"[Title/Abstract] OR "age related maculopathies"[Title/Abstract] OR "age related maculopathies"[Title/Abstract] OR "age related maculopathy"[Title/Abstract] OR "age related maculopathy"[Title/Abstract] OR "Macular Degeneration"[MeSH Terms]) AND ("learning deep"[Title/Abstract] OR "hierarchical learning"[Title/Abstract] OR "learning hierarchical"[Title/Abstract] OR "Deep Learning"[MeSH Terms]) | 141 | 7:59:05 |

**(b) EMBASE search history**

| No. | Query | Results | Date |
| --- | --- | --- | --- |
| #28 | #5 AND #27 | 352 | 8-Dec-22 |
| #27 | #6 OR #7 OR #8 OR #9 OR #10 OR #11 OR #12 OR #13 OR #14 OR #15 OR #16 OR #17 OR #18 OR #19 OR #20 OR #21 OR #22 OR #23 OR #24 OR #25 OR #26 | 46791 | 8-Dec-22 |
| #26 | 'age related maculopathy':ti,ab | 957 | 8-Dec-22 |
| #25 | 'age-related maculopathy':ti,ab | 957 | 8-Dec-22 |
| #24 | 'age related maculopathies':ti,ab | 11 | 8-Dec-22 |
| #23 | 'age-related maculopathies':ti,ab | 11 | 8-Dec-22 |
| #22 | 'maculopathy, age related':ti,ab | 4 | 8-Dec-22 |
| #21 | 'maculopathy, age-related':ti,ab | 4 | 8-Dec-22 |
| #20 | 'maculopathies, age-related':ti,ab | 1 | 8-Dec-22 |
| #19 | 'macular degeneration, age related':ti,ab | 39 | 8-Dec-22 |
| #18 | 'macular degeneration, age-related':ti,ab | 39 | 8-Dec-22 |
| #17 | 'age-related macular degenerations':ti,ab | 25 | 8-Dec-22 |
| #16 | 'age related macular degeneration':ti,ab | 28457 | 8-Dec-22 |
| #15 | 'age-related macular degeneration':ti,ab | 28464 | 8-Dec-22 |
| #14 | 'macular dystrophies':ti,ab | 287 | 8-Dec-22 |
| #13 | 'dystrophy, macular':ti,ab | 27 | 8-Dec-22 |
| #12 | 'macular dystrophy':ti,ab | 1885 | 8-Dec-22 |
| #11 | 'maculopathies':ti,ab | 354 | 8-Dec-22 |
| #10 | 'maculopathy':ti,ab | 6437 | 8-Dec-22 |
| #9 | 'macular degenerations':ti,ab | 168 | 8-Dec-22 |
| #8 | 'degeneration, macular':ti,ab | 54 | 8-Dec-22 |
| #7 | 'macular degeneration':ti,ab | 32840 | 8-Dec-22 |
| #6 | 'age related macular degeneration'/exp | 26146 | 8-Dec-22 |
| #5 | #1 OR #2 OR #3 OR #4 | 32183 | 8-Dec-22 |
| #4 | 'learning, hierarchical':ti,ab | 48 | 8-Dec-22 |
| #3 | 'hierarchical learning':ti,ab | 100 | 8-Dec-22 |
| #2 | 'learning, deep':ti,ab | 330 | 8-Dec-22 |
| #1 | 'deep learning'/exp | 31916 | 8-Dec-22 |

**(c) the Cochrane Library**

#1 MeSH descriptor: [Deep Learning] explode all trees 77

#2 Learning, Deep 1393

#3 Hierarchical Learning 335

#4 Learning, Hierarchical 335

#5 #1 OR #2 OR #3 OR #4 1694

#6 MeSH descriptor: [Macular Degeneration] explode all trees MeSH 2848

#7 Age-Related Maculopathies

#8 Age Related Macular Degeneration 3463

#9 Age-Related Maculopathy 168

#10 Macular Degeneration, Age-Related 3326

#11 Age-Related Macular Degenerations 3

#12 Age Related Maculopathy 249

#13 Maculopathy, Age Related 249

#14 Age-Related Macular Degeneration 3326

#15 Age Related Maculopathies 6

#16 Macular Degeneration, Age Related 3463

#17 Maculopathies, Age-Related 3

#18 Maculopathy, Age-Related 168

#19 Maculopathies; Dystrophy, Macular 0

#20 Maculopathy 465

#21 Macular Dystrophies 18

#22 Macular Degenerations 17

#23 Degeneration, Macular 3805

#24 Macular Dystrophy 60

#25 #6 OR #7 OR #8 OR #9 OR #10 OR #11 OR #12 OR #13 OR #14 OR #15 OR #16 OR #17 OR #18 OR #19 OR #20 OR #21 OR #22 OR #23 OR #24 5349

#26 #5 AND #25 26

**(d) ScienceDirect**

Title, abstract or author-specified keywords:

((macular degeneration*) OR (degeneration, macular) OR (maculopathy) OR (maculopathies) OR (dystrophy, macular) OR (macular dystrophies) OR (age related maculopathy)) AND ((learning, deep) OR (hierarchical learning)

**(e) Scopus**

( ( 'age AND related AND maculopathy':ti,ab ) OR ( 'age-related AND maculopathy':ti,ab ) OR ( 'age AND related AND maculopathies':ti,ab ) OR ( 'age-related AND maculopathies':ti,ab ) OR ( 'maculopathy, AND age AND related':ti,ab ) OR ( 'maculopathy, AND age-related':ti,ab ) OR ( 'maculopathies, AND age-related':ti,ab ) OR ( 'macular AND degeneration, AND age AND related':ti,ab ) OR ( 'macular AND degeneration, AND age-related':ti,ab ) OR ( 'age-related AND macular AND degenerations':ti,ab ) OR ( 'age AND related AND macular AND degeneration':ti,ab ) OR ( 'age-related AND macular AND degeneration':ti,ab ) OR ( 'macular AND dystrophies':ti,ab ) OR ( 'dystrophy, AND macular':ti,ab ) OR ( 'macular AND dystrophy':ti,ab ) OR ( 'maculopathies':ti,ab ) OR ( 'maculopathy':ti,ab ) OR ( 'macular AND degenerations':ti,ab ) OR ( 'degeneration, AND macular':ti,ab ) OR ( 'macular AND degeneration':ti,ab ) OR ( 'age AND related AND macular AND degeneration'/exp ) ) AND ( ( 'learning, AND hierarchical':ti,ab ) OR ( 'hierarchical AND learning':ti,ab ) OR ( 'learning, AND deep':ti,ab ) OR ( 'deep AND learning'/exp ) )

(**f)Web of Science**

(((((((((((((((((((((KP=("age related macular degeneration")) OR AB=(macular degeneration)) OR AB=(degeneration, macular)) OR AB=(macular degenerations)) OR AB=(maculopathy)) OR AB=(maculopathies)) OR AB=(macular dystrophy)) OR AB=(dystrophy, macular)) OR AB=(macular dystrophies)) OR AB=(age-related macular degeneration)) OR AB=(age related macular degeneration)) OR AB=(age-related macular degenerations)) OR AB=(macular degeneration, age-related)) OR AB=(macular degeneration, age related)) OR AB=(maculopathies, age-related)) OR AB=(maculopathy, age-related)) OR AB=(maculopathy, age related)) OR AB=(age-related maculopathies)) OR AB=(age related maculopathies)) OR AB=(age-related maculopathy)) OR AB=(age related maculopathy)) AND (((AB=(learning, deep)) OR AB=(hierarchical learning)) OR AB=(learning, hierarchical)) OR KP=("deep learning"))
